# EpiInsula: calibrated regression of the Hi-C insulation score from cell-type-specific epigenomic signal

**DOI:** 10.1093/bib/bbag589

**Published:** 2026-10-26

**Authors:** Yuyu Jin, Tong Chen, Jie Gao, Mengyao Tong, Guangcheng Xu, Xueyu Zhang, Weixu Liu, Yin Shen

**Affiliations:** School of Biomedical Engineering, Anhui Medical University, 81 Meishan Road, Shushan District, Hefei, Anhui 230032, China; School of Biomedical Engineering, Anhui Medical University, 81 Meishan Road, Shushan District, Hefei, Anhui 230032, China; School of Biomedical Engineering, Anhui Medical University, 81 Meishan Road, Shushan District, Hefei, Anhui 230032, China; School of Biomedical Engineering, Anhui Medical University, 81 Meishan Road, Shushan District, Hefei, Anhui 230032, China; School of Biomedical Engineering, Anhui Medical University, 81 Meishan Road, Shushan District, Hefei, Anhui 230032, China; School of Biomedical Engineering, Anhui Medical University, 81 Meishan Road, Shushan District, Hefei, Anhui 230032, China; School of Medical Humanities, Anhui Medical University, 81 Meishan Road, Shushan District, Hefei, Anhui 230032, China; School of Biomedical Engineering, Anhui Medical University, 81 Meishan Road, Shushan District, Hefei, Anhui 230032, China

**Keywords:** chromatin organisation, insulation score, Hi-C, epigenomic prediction, deep learning, cross-cell-type transfer

## Abstract

Chromatin insulation shapes which regulatory elements can reach which promoters, but much computational prediction of chromatin organisation has been posed as boundary classification, which discards the depth and shape of the insulation profile. We developed EpiInsula, a dilated convolutional framework that predicts the 10-kb Hi-C insulation score as a continuous quantity from six cell-type-specific epigenomic tracks read over a ±700-kb window. Under a chromosome-disjoint partition that scores every evaluated locus with a model which never saw its chromosome, held-out correlations across four human cell lines are 0.791, 0.780, 0.860 and 0.774, and the regression of measured on predicted insulation has slopes of 0.90–0.95, indicating calibration of the conditional mean, the sense used throughout. Against seven methods fitted under a single evaluation contract, EpiInsula gives the highest correlation and, with boundary thresholds selected on validation chromosomes and frozen before testing, the highest boundary F1 against 4D Nucleome calls in every cell line; the lead is retained on the fine-scale component of the profile, and three published contact-map predictors retrained on the same data give lower correlation on the derived insulation score. On boundaries that differ between IMR-90 and K562 it recovers 0.383 of the contrasts against 0.024–0.264 for all seven comparison methods; it ranks first at every point of a 25-point threshold grid and in all six cell-line pairs, and the remaining methods are ranked differently from pooled correlation. EpiInsula provides a framework for predicting chromatin organisation on a continuous scale from routinely available epigenomic assays. Availability: https://github.com/Yin-Shen/EpiInsula

## Introduction

The 3D organisation of chromatin within the nucleus constitutes a fundamental layer of gene regulation [1, 2]. Chromosome conformation capture has revealed that mammalian genomes are partitioned into self-interacting regions, designated topologically associating domains [3, 4]. Within such a domain, positions contact one another far more frequently than they contact neighbouring regions. The positions at which these domains meet, commonly termed boundaries, constrain which regulatory elements can reach which promoters. Disruption of boundary integrity can rewire enhancer–promoter communication and activate genes that are normally insulated, contributing to limb malformation and to oncogene activation in glioma and in T-cell leukaemia [5–7]. It is therefore important to determine where domains are separated and how strongly.

The strength of separation at a given position is commonly quantified by the insulation score [8, 9]. It is computed by sliding a square window along the diagonal of the contact matrix and summing the contacts that cross each position. The insulation score is a continuous quantity defined at every genomic bin, and boundaries are conventionally called as its local minima. To measure the contact frequencies from which it is derived, researchers have developed chromosome conformation capture and its derivatives, including 4C, 5C, and chromatin interaction analysis by paired-end tag sequencing [10–13]. Genome-wide approaches followed, including Hi-C and, at nucleosome resolution, Micro-C [14–16]. However, resolving insulation at 10-kb bins requires deep sequencing of each sample, because the interaction space grows with the square of the number of bins, so that sequencing requirements rise faster than linearly as the bin size is reduced [17–19]. Reliable computational prediction could therefore extend insulation profiling to the many cell types for which contact maps are not available.

The epigenomic determinants of domain organisation provide a natural basis for such prediction. Loop extrusion by the cohesin complex, halted at convergently oriented CTCF sites, accounts for the position and orientation of many domain boundaries [20, 21], and the extrusion activity of human cohesin has since been observed directly [22]. Targeted degradation of CTCF removes local insulation while leaving compartmental organisation largely intact [23]. CTCF occupancy, cohesin binding and chromatin accessibility are measured by assays far less costly than Hi-C, and are already available for hundreds of cell types. A mapping from these signals to chromatin organisation is therefore both biologically motivated and practically attractive. Computational methods have accordingly been developed to predict domain organisation from one-dimensional signal. preciseTAD applies transfer learning to boundary prediction at base-pair resolution [24], while Epiphany predicts contact maps from epigenomic tracks with an adversarial objective [25]. C.Origami combines sequence with cell-type-specific signal to predict cell-type-specific organisation [26], and ChromNet reads the same inputs with multi-task training aimed at cross-cell-type prediction [27]. Related approaches predict interaction matrices or contacts from combinations of sequence and epigenomic features [28, 29].

A parallel line of work predicts chromatin organisation from DNA sequence alone. Akita predicts contact maps directly from sequence [30], Orca extends this to multiple scales [31], and DeepC transfers megabase-scale representations across tasks [32]. Dilated convolutions reach distal signal without a corresponding increase in parameters, and were introduced for this purpose in sequential regulatory-activity prediction [33]; subsequent work reaches longer ranges with self-attention instead [34]. Despite these advances, a substantial line of this work formulates prediction of domain organisation as boundary classification, in which a genomic bin is labelled as a boundary or not [24, 35, 36]. That formulation discards the depth and shape of the insulation profile, retaining only the positions of its minima. It also makes methods difficult to compare, because each study defines its boundary labels differently. Reported accuracies therefore depend on target definitions and evaluation data that differ between papers, and the reference domains themselves vary with the caller, the resolution, and the normalisation used to derive them [37, 38]. A further consequence concerns what aggregate accuracy measures. Much of the domain landscape is conserved across cell types [3, 39], so that shared component dominates any pooled score. A method can therefore score well while contributing little at the positions where cell types actually differ.

To address these challenges, we developed EpiInsula, a dilated convolutional framework that predicts the insulation score as a continuous quantity from six cell-type-specific epigenomic tracks read over a ±700-kb window. The framework makes four contributions. (i) It poses chromatin organisation prediction as calibrated regression rather than boundary classification, with calibration meant in the conditional-mean sense defined in Methods. The network is assembled from established components, a dilated residual convolution with a centre-plus-global read-out, and no architectural novelty is claimed; the contribution lies in the target, the evaluation design, and the characterisation of the fitted model. Evaluation uses a chromosome-disjoint partition, in which each of the 973 169 evaluated autosomal loci is scored by a model that never saw its chromosome. (ii) It is compared against seven methods under a single contract that holds the input tracks, the window, the folds, the seeds, and the held-out loci fixed, so that reported differences reflect the models rather than their evaluation, and against three published contact-map predictors retrained with their own code and inputs on the same Hi-C data. (iii) It is evaluated on an endpoint defined only where two cell types disagree, in addition to aggregate accuracy, which distinguishes prediction of the shared landscape from prediction of cell-type-specific organisation. (iv) Input-group ablation and cross-cell-type transfer characterise which signals the prediction draws upon, and how much of it survives a change of cell type with and without labels from the target. Together these contributions provide a framework for predicting chromatin organisation on a continuous scale and for evaluating such predictions where cell types differ.

## Materials and methods

### Overview of the prediction task and pipeline

EpiInsula predicts the 10-kb Hi-C insulation score from six cell-type-specific epigenomic tracks over a ±700-kb window (Fig. 1A). The task is continuous regression rather than binary boundary calling. Separate models are fitted for each cell line, and cell specificity is carried by the input tracks. Figure 1D summarises the held-out analyses (Figs 2–6); complete architecture and training settings are provided in Supplementary Methods.

**Figure 1:**
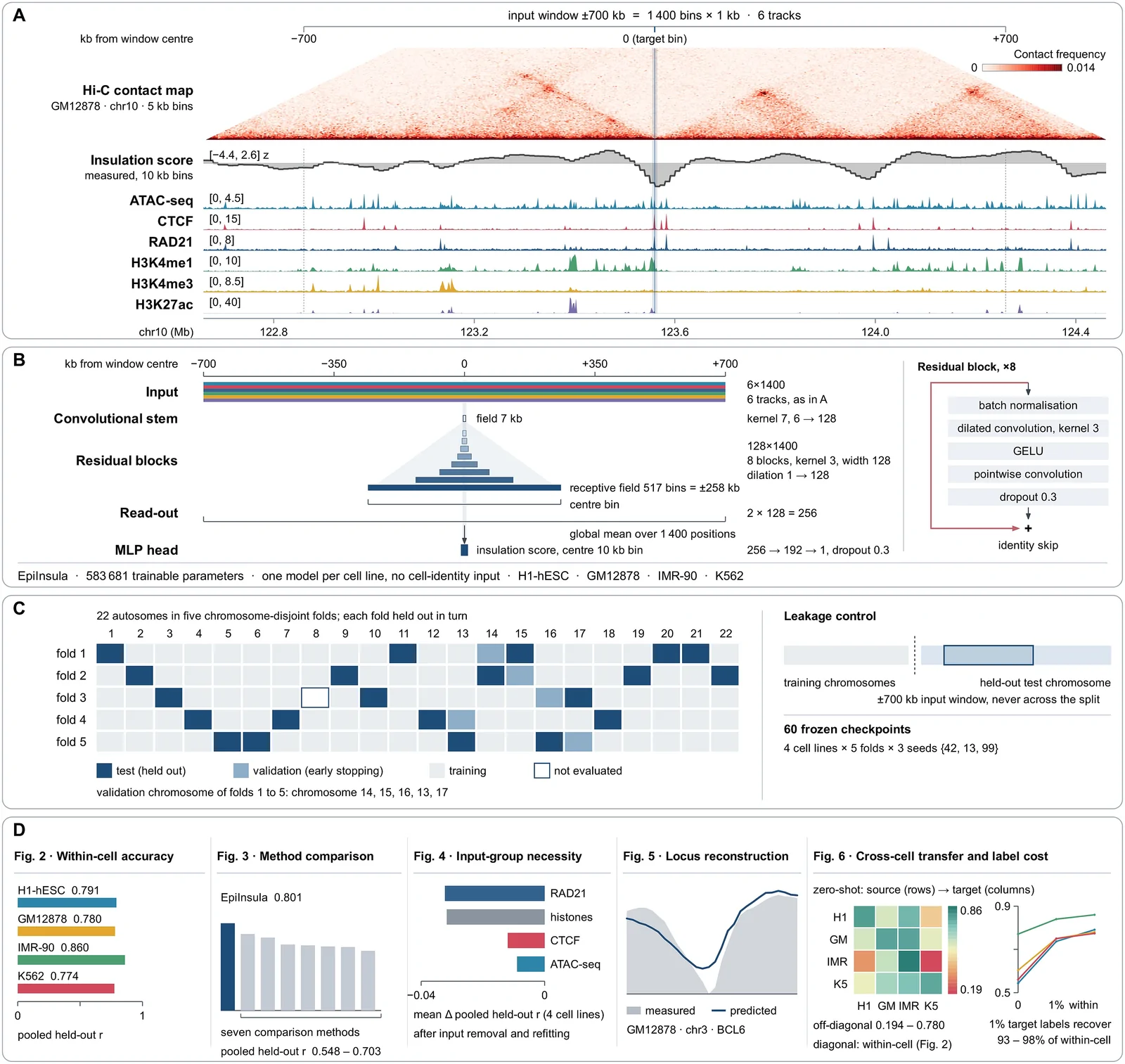
**Task, model and chromosome-disjoint evaluation.** (A) Measured GM12878 data over chr10:122.66–124.46 Mb: 4D Nucleome Hi-C, the measured insulation profile and six epigenomic inputs. The central 10-kb target bin and ±700-kb input window are marked. (B) EpiInsula architecture. True bar widths depict the widening receptive field; the read-out combines central-position features with the global mean. (C) Five whole-chromosome folds, validation chromosomes, and the 60 cell-line × fold × seed checkpoints; chromosome 8, on which the input window was selected, is not evaluated. (D) Values from the analyses in Figs 2–6: within-cell prediction, method comparison, input-group necessity, illustrative BCL6 reconstruction at GM12878 chr3:187.60–187.88 Mb (Fig. 5B) and cross-cell transfer. The input-group summary is the unweighted mean across the four cell-line-specific changes in seedwise pooled held-out raw-scale Pearson correlation after removal and refitting, as reported in Fig. 4B and Supplementary Table S13. The transfer summary uses the validation-selected source of Fig. 6C; the full source–target matrix is shown in Fig. 6A.

**Figure 2:**
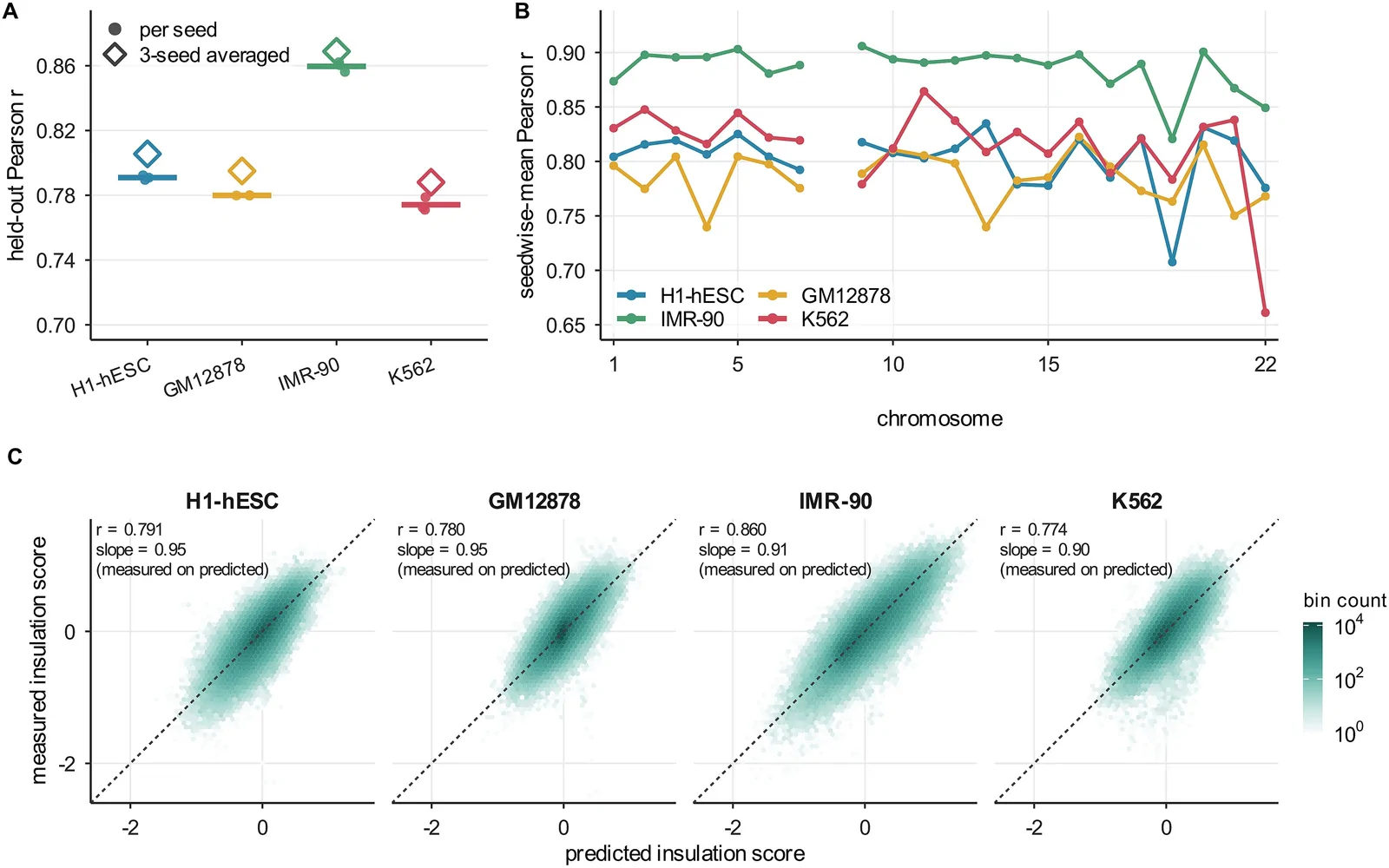
**Within-cell accuracy, chromosome spread, and amplitude.** (A) Held-out Pearson correlation. Horizontal rules are seedwise means; filled circles are seeds 42, 13, and 99; open diamonds score the three-seed averaged prediction. The axis begins at 0.70 and no point falls below it. (B) Seedwise-mean correlation for each evaluated autosome, scored by the fold that held it out; chromosome 8 is not evaluated. (C) Seed-42 hexbin density of measured versus predicted insulation on pooled held-out loci (H1-hESC, *n* = 241 078; GM12878, *n* = 244 234; IMR-90, *n* = 243 101; K562, *n* = 244 756). The dashed rule is identity. Displayed Pearson correlations and slopes of measured on predicted insulation are means of the three seed-specific metrics, rather than statistics recomputed from the displayed seed-42 cloud. Colour is logarithmic bin count.

**Figure 3:**
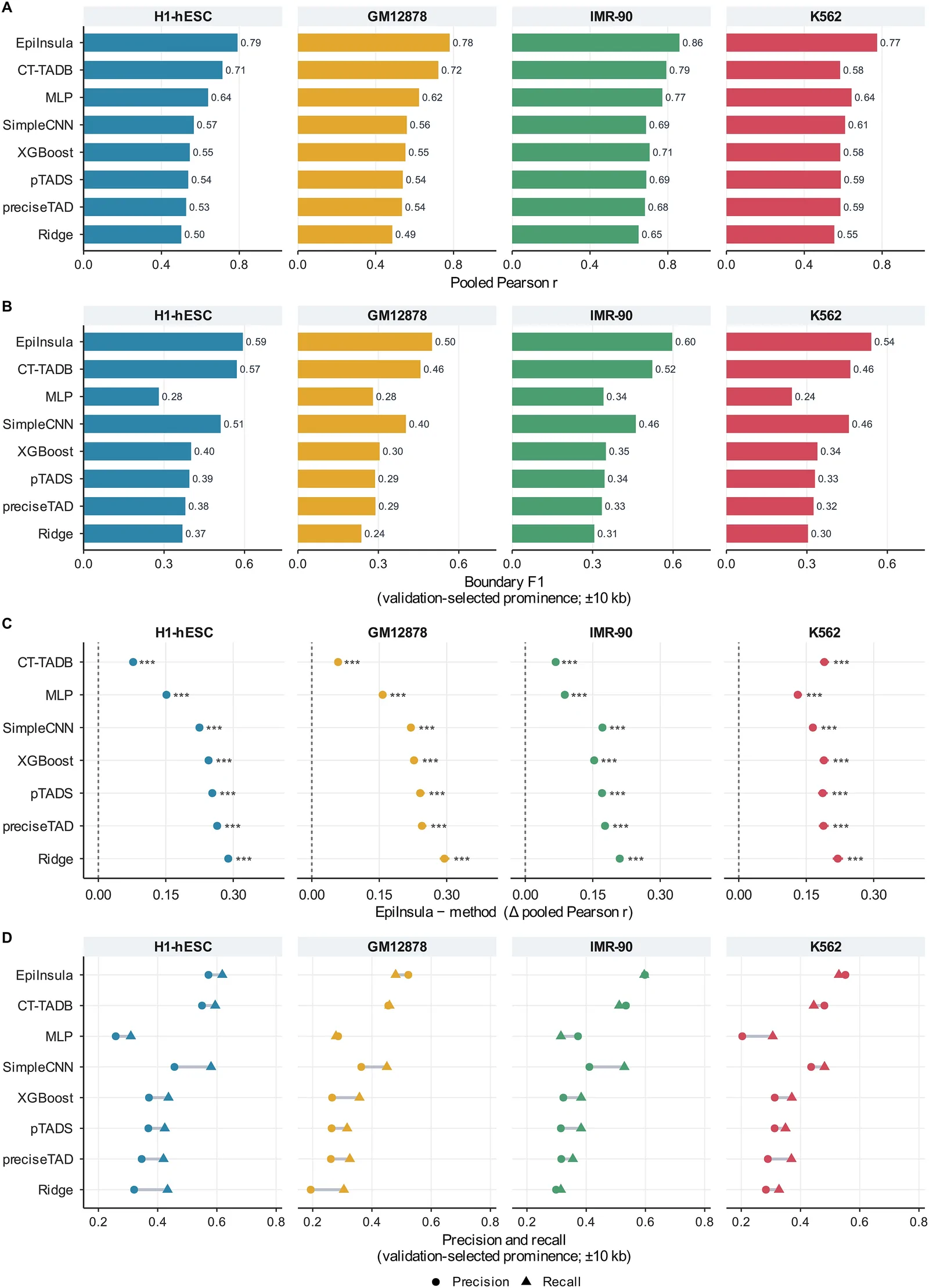
**Held-out performance of EpiInsula and seven comparison models.** All models used the same six tracks, ±700-kb window, five folds, three seeds, and held-out loci. CT-TADB, preciseTAD, and pTADS are task-adapted implementations, not reproductions of the published methods; Supplementary Methods describes each implementation. (A) Seedwise-mean pooled held-out correlation after decoding every fold with its training-set target scaler before pooling. (B) Boundary F1 within ±10 kb against the 4D Nucleome reference calls (Materials and methods), with the prominence selected on each fold’s validation chromosome (Supplementary Table S7); bars are seedwise means. Exact-bin and ±20-kb matching are shown in Supplementary Fig. S1. (C) Paired correlation differences between EpiInsula and each comparison model, with 95% gap-aware block-bootstrap intervals taken as the union over 50-, 100-, and 200-bin blocks. One-sided probabilities are the largest across block lengths and are Benjamini–Hochberg corrected over 28 tests. Triple asterisks denote adjusted *P* < .001. (D) Precision (circles) and recall (triangles) within ±10 kb at the prominence of panel B; seedwise means, values in Supplementary Table S7.

**Figure 4:**
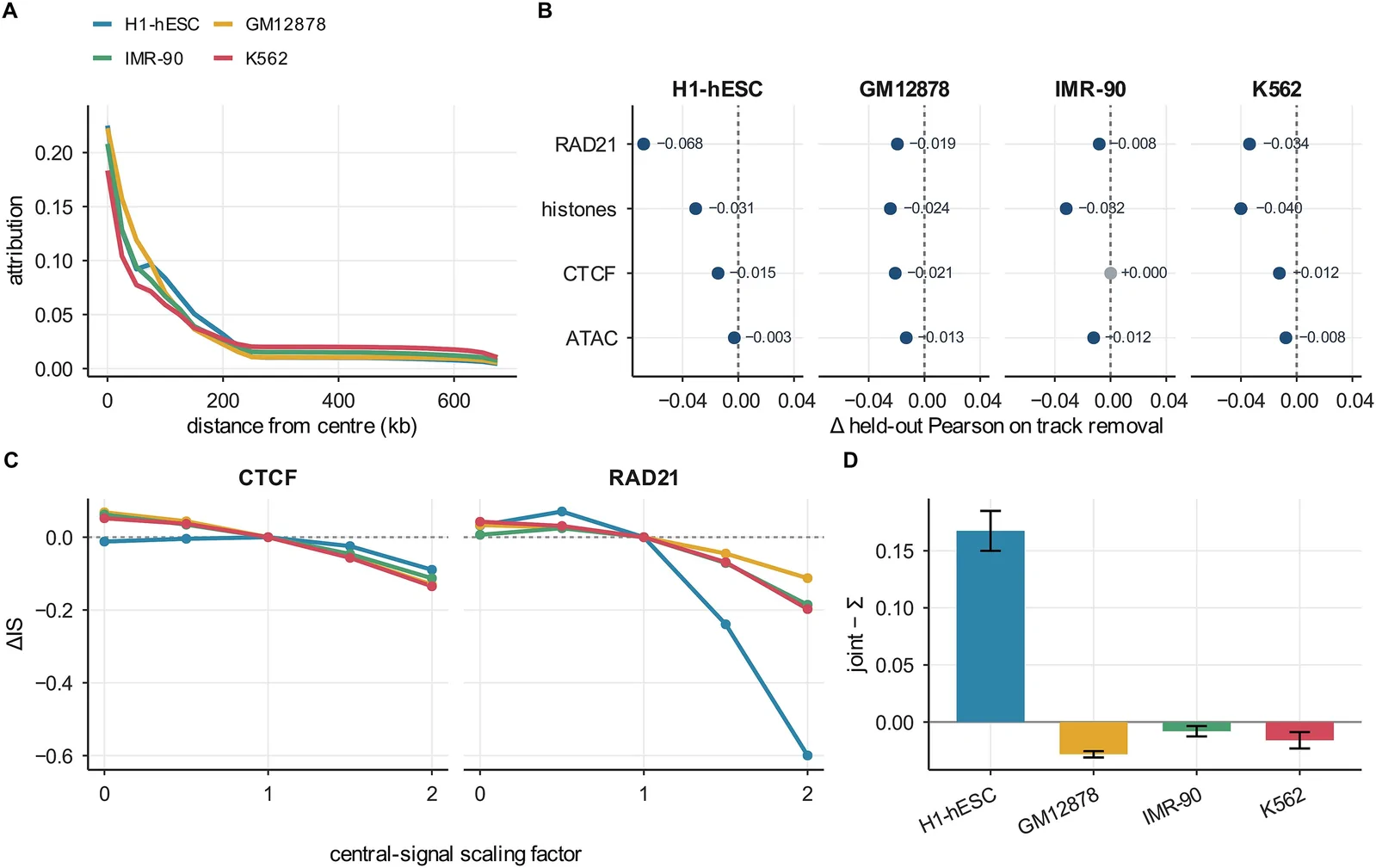
**Input-group necessity and inference-time response.** (A) Mean absolute gradient × input versus absolute distance from the window centre, binned at 25 kb on held-out loci at seed 42. Each fold is normalised to sum to one across 28 distance bins; curves are fold means and bands are mean ± 2 standard errors over *n* = 5 folds. (B) Change in held-out correlation after removing CTCF, RAD21, ATAC-seq, or all three histone tracks and refitting from random initialisation (240 fits). Error bars are unadjusted 95% gap-aware 200-bin block-bootstrap intervals, and the change is printed beside each interval. Grey denotes an interval spanning zero. (C) Change in predicted insulation after multiplying the central ±20 kb of transformed CTCF or RAD21 signal by 0, 0.5, 1.5, or 2.0. Bands are standard errors over pooled held-out loci. Factor zero sets the median-centred slice to its training median. (D) Joint CTCF-and-RAD21 attenuation minus the sum of single-channel responses. Bars are fold means; error bars are the population standard deviation across five folds divided by √5 at seed 42. Panels A, C, and D probe the fitted function and do not establish chromatin mechanisms.

**Figure 5:**
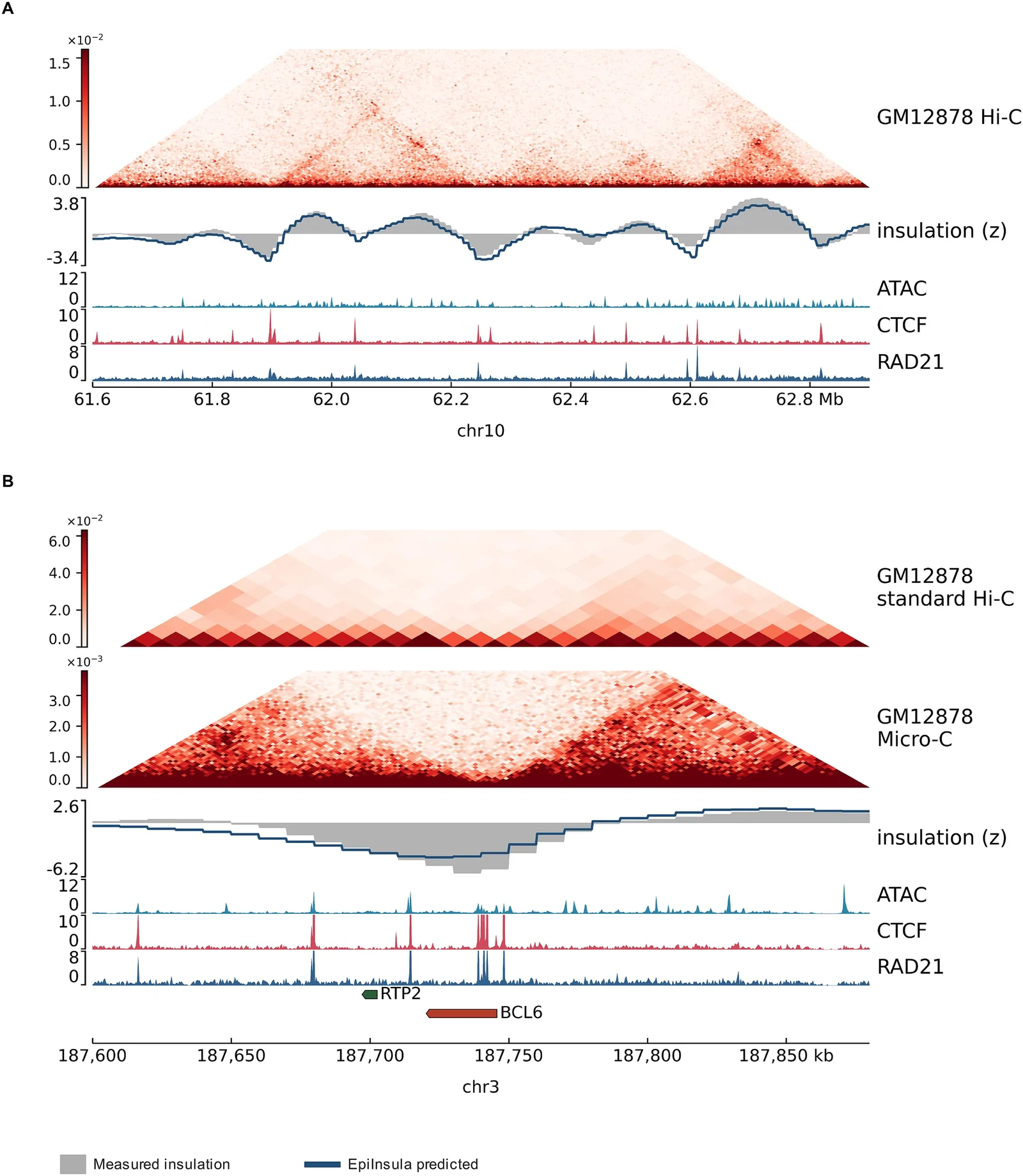
**Profile reconstruction at two illustrative held-out loci.** The loci shown are illustrative examples from the held-out chromosomes and are not treated as statistical replicates; quantitative conclusions are based on the aggregate held-out evaluations reported in the corresponding panels and supplementary analyses. Grey areas are measured insulation; navy lines are EpiInsula predictions. ATAC-seq, CTCF, and RAD21 are model inputs, so co-localisation describes model context rather than independent validation. (A) A multi-domain GM12878 window, chr10:61.6–62.9 Mb, with 4D Nucleome Hi-C and three input tracks. (B) The BCL6 region, chr3:187.60–187.88 Mb, with standard Hi-C and higher-resolution Micro-C on the same coordinates. Micro-C provides a higher-resolution descriptive view of the same cell type and was not used for model training. Contact maps show balanced contact frequency and are scaled independently.

**Figure 6:**
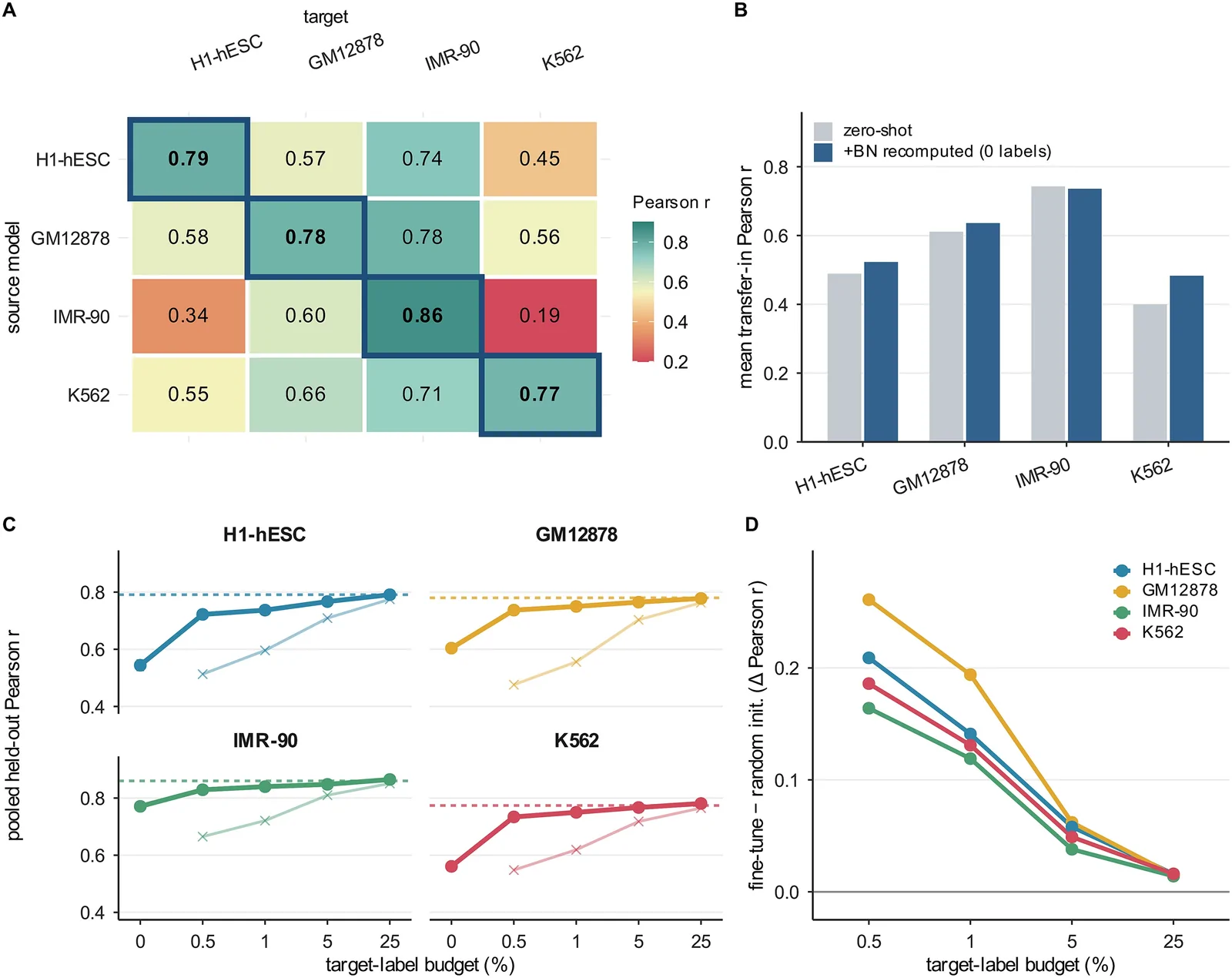
**Transfer to an unseen cell line and its label cost.** (A) Seedwise-mean correlation for all 16 ordered source–target pairs without weight updates, with target inputs standardised by the target’s own scaler. Outlined diagonal entries reproduce within-cell performance and are not transfer. (B) Absolute mean transfer-in correlation before and after recomputing target batch-normalisation statistics without labels or weight updates. Each bar averages the three source cell lines for that target; no inferential error bar is shown. (C) Held-out correlation versus target-label budget. Filled circles and solid lines are source-initialised fine-tuning; light crosses are random initialisation on the same subset; dashed lines are within-cell values. Fine-tuning error bars are ± population standard deviation over *n* = 3 seeds; scratch curves have no error bars. At 0%, only the source-initialised arm is defined. (D) Fine-tuning minus random initialisation at matched non-zero budgets, computed from the seedwise-mean curves; no interval is shown. Budgets are equally spaced categories. Panels C and D use, for each fold, the source selected by zero-shot correlation on that fold’s validation chromosome; panel A shows all source–target pairs without source selection.

### Cell lines and input data sources

Four GRCh38 human cell lines were modelled: H1-hESC, GM12878, IMR-90, and K562. Public epigenomic inputs and insulation targets were obtained from ENCODE [40–42] and the 4D Nucleome [43–46]; per-file accessions, assemblies, and provenance are listed in Supplementary Table S1.

The six inputs are ATAC-seq, CTCF, RAD21, H3K4me1, H3K4me3, and H3K27ac. All are cell-type-specific processed signal tracks; sequence-derived channels are not used.

### Insulation score as the prediction target

The target is the diamond insulation score. For bin *i* and half-window *w*, *S*(*i*, *w*) is the mean balanced contact frequency between the w upstream and w downstream bins. The reported score is *S* normalised by the median over the mappable region of the corresponding chromosome arm and transformed on a base-2 logarithmic scale,


(1) \begin{equation*} IS\left(i,w\right)={\mathit{\log}}_2\ \left[S\left(i,w\right)/{median}_{arm}\ S\left(\cdotp, w\right)\right] \end{equation*}

so values near zero indicate typical across-bin contact and increasingly negative values indicate stronger insulation.

Released 4D Nucleome insulation tracks [46], generated with cooltools [47], were aggregated to the 10-kb prediction grid. Fold-specific target standardisation was fitted on training chromosomes and inverted before regression metrics were calculated; full processing details are given in Supplementary Methods.

### Locus definition and quality filtering

A locus is one 10-kb target bin with its surrounding epigenomic window. It is admitted when the target and all six signals meet the finite-coverage criteria and the stored ±1-Mb window lies within the chromosome; remaining non-finite signal values in admitted windows are set to zero. Filtering is applied during dataset construction, as detailed in Supplementary Methods.

The filter admits 254 834, 258 012, 256 850 and 258 635 autosomal loci in H1-hESC, GM12878, IMR-90, and K562, respectively (1 028 331 total; Supplementary Table S2).

### Input representation and signal normalisation

Signals are binned at 1 kb, and the model reads a 6 × 1400 matrix spanning ±700 kb around each locus. The half-width is a development hyperparameter, selected, before the five-fold cross-validation, on a development partition whose validation chromosome, chromosome 8, is excluded from every held-out evaluation (Supplementary Table S3). The network was trained at seven half-widths from ±100 to ±1000 kb, each a centre-crop of the same stored ±1-Mb inputs, and the half-width retained was the smallest whose validation correlation, averaged over the four cell lines and three seeds, lay within 0.001 of the highest average.

Within each cell line and fold, robust input scaling and target standardisation are fitted on training chromosomes only. Scaled signals undergo an inverse hyperbolic sine transform and clipping at ±30. Targets are decoded before regression metrics are computed; sampling and transformation details are provided in Supplementary Methods.

### Network architecture and receptive field

EpiInsula is a one-dimensional dilated residual convolutional network (Fig. 1B). A width-7 stem maps the six channels to 128 features, followed by eight residual blocks with width-3 convolutions at dilations 1–128, batch normalisation, Gaussian error linear units, pointwise convolutions, dropout, and identity skips.

The read-out combines central-position features with the global mean over the window and predicts one score through a two-layer perceptron. The model has 583 681 trainable parameters; the receptive-field calculation and parameter decomposition are given in Supplementary Table S4.

### Training objective and optimisation settings

The objective combines smooth *L*1 loss on the standardised target with a within-batch correlation term,


(2) \begin{equation*} L= Smooth\,L1\left(\hat{\mathrm{y}},y\right)+0.3\left(1-r\right) \end{equation*}

where β = 1 and the correlation term has weight 0.3. The correlation term is one minus the Pearson correlation between the predictions and the standardised targets of a batch and is invariant to a shift or a positive rescaling of the predictions.

Models use AdamW, a two-epoch warm-up and cosine decay over a 60-epoch budget, with early stopping on validation correlation. Batch size, regularisation, augmentation, precision settings, and software versions are reported in Supplementary Methods and Supplementary Table S4.

### Cross-validation design and information-leakage control

All partitioning is by whole chromosome to prevent nearby correlated loci from appearing in both training and test sets.

The 22 autosomes are divided into five predefined test blocks, each with a separate validation chromosome; the remaining chromosomes train the fold (Fig. 1C). Exact chromosome assignments are listed in Supplementary Methods.

Input scalers, target standardisation, weights, and early-stopping decisions use only the training and validation chromosomes of the corresponding fold.

Each cell line and fold is fitted with seeds 42, 13, and 99, giving 60 primary models. Concatenating the five test blocks yields one held-out prediction per admitted locus and seed. Chromosome 8, on which the input half-width was selected, is excluded from every held-out evaluation, so that reported results cover 973 169 loci on 21 autosomes and fold 3 is scored on chromosomes 3, 10, and 17. Comparison, ablation and transfer analyses reuse the same fold geometry.

### Evaluation metrics, estimands, and dispersion

The primary metric is Pearson correlation on the decoded held-out series. Calibration is summarised by ordinary-least-squares regression of measured values y on predictions ŷ,


(3) \begin{equation*} y=a+b\ \hat{y} \end{equation*}

Throughout, ‘calibrated’ refers to calibration of the conditional mean: the mean measured value among loci with a given prediction equals that prediction. It is assessed by the slope and intercept of this regression, which are one and zero when this holds. The term does not refer to calibrated uncertainty. A slope near one indicates calibration of the conditional mean but does not imply recovery of each locus’s measured amplitude. Reverse regression and identity-line R^2^ quantify amplitude compression and absolute agreement.

Depth recovery summarises the predicted-to-measured mean among the deepest 1% of measured insulation values; definitions of all secondary metrics are provided in Supplementary Methods.

Metrics are calculated per seed on pooled held-out predictions and then averaged, except where prediction averaging or seed-42 inference probes are explicitly stated. Seed dispersion is the sample standard deviation over three seeds.

### Uncertainty quantification by genomic block bootstrap

Uncertainty intervals use chromosome-stratified, gap-aware block bootstrap resampling with 2000 draws. Paired comparisons reuse the same draws. Ablations use 200-bin blocks; method comparisons conservatively combine 50-, 100-, and 200-bin results and apply Benjamini–Hochberg correction across 28 tests. Full definitions are provided in Supplementary Methods.

These intervals describe locus-level variation conditional on the observed chromosome set; fold-level spread is reported separately.

### Comparison methods and the task-matched contract

Seven alternatives are evaluated: task-adapted regressors based on CT-TADB [36] (its published convolutional–Transformer hybrid encoder, with the six tracks in place of its native inputs and a regression output in place of the classification head), preciseTAD [24] and pTADS [48]; a multilayer perceptron and plain convolutional network; ridge regression; and gradient-boosted trees. The adaptations retain the published architectures and learners but change the target from a boundary label to the continuous insulation score and do not reproduce the original methods or their reported performance. Feature summaries are detailed in Supplementary Methods; architectures, fixed settings, search spaces and the values selected over the fits are given in Supplementary Table S5.

Every method uses the same inputs, window, chromosome folds, seeds, and held-out loci. Hyperparameters of the tree and linear methods are selected on the fold’s validation chromosome; the networks use one fixed recipe. The preciseTAD and pTADS adaptations use capped training subsamples, while all methods predict the complete held-out split.

### Comparison with contact-map prediction methods

Three published methods that predict the contact map rather than the insulation score, Epiphany [25], C.Origami [26] and ChromNet [27], were run in two settings; Supplementary Table S6 sets them against EpiInsula by input, output, resolution, training target, and training chromosomes. In the first setting each method was retrained with its authors’ code and native inputs on our Hi-C matrices under fold 1 of the partition (validation chromosome 14; test chromosomes 1, 11, 15, 20, and 21), one fit per cell line at seed 42. Epiphany reads five signal tracks at 100-bp resolution (DNase-seq, H3K27ac, H3K27me3, H3K4me3, and CTCF) and predicts a 1-Mb diagonal band at 10-kb resolution from targets built with HiC-DC+ from our contact matrices. Its training loop was re-implemented around the authors’ unchanged model, dataset, loss, optimiser, and schedule; relative to the released script, the chromosome split, the scoring of the full validation set each epoch, the retention of the validation-optimal checkpoint, the seed handling, and the data-loading paths were changed, and early stopping at patience 12 was added within the 55-epoch budget. C.Origami and ChromNet read DNA sequence with CTCF and ATAC-seq and predict a 2.1-Mb square at 8192-bp resolution. Both were trained under their released 80-epoch schedule with the validation-optimal checkpoint retained, on one GPU at the released per-device batch size of 8 (the released configuration uses four GPUs), after modifications for the chromosome split and library-version compatibility; the number of data-loading workers was reduced. In the second setting the weights released by each group, one checkpoint per method (Epiphany fitted on GM12878; C.Origami and ChromNet on IMR-90), were applied to each cell line’s inputs without retraining; those checkpoints were fitted on chromosome sets that overlap the test chromosomes, as recorded in Supplementary Table S6. In both settings the insulation score was derived from the predicted map in its native output domain and resolution by the steps of the target definition. Contacts were returned to the linear domain, the diamond mean was taken at a half-width of 100 kb (98 304 bp on the 8192-bp grid), overlapping windows were averaged and the result was log2-transformed. The profile was resampled to the 10-kb grid, the log2 median over the mappable bins of the chromosome arm was subtracted, and the profile was correlated with the measured score over the five test chromosomes. For Epiphany the map is the HiC-DC+-normalised band on which the method is trained; for C.Origami and ChromNet it is the predicted balanced map. EpiInsula is reported on the same fold, seed, and chromosomes.

### Boundary calling and cell-type-specific boundaries

Reference boundaries are released 4D Nucleome calls associated with the target workflow [49] (Supplementary Table S1). Strong and Weak calls are retained, assigned to the 10-kb grid and deduplicated.

Predicted boundaries are local minima of the per-fold standardised profile, located by topographic prominence on the negated profile within contiguous runs of 10-kb bins. For each method, fold, cell line, and seed, the prominence is selected on the fold’s validation chromosome from the common grid 0.05, 0.1, 0.15, 0.2, 0.3, 0.4, 0.5, 0.6, 0.8, 1.0, 1.2, 1.5, and 2.0 as the smallest grid value that maximises F1 within ±10 kb against the reference calls there, and is then frozen. Boundaries are called on the fold’s test chromosomes at that value; true positives, false positives, and false negatives are accumulated over the five folds, and F1 is formed once per seed and averaged over the three seeds. The procedure is identical across methods. A predicted and a reference boundary match when their bins lie no more than one 10-kb bin apart; matching at the exact bin and within two bins (±20 kb) is reported as a sensitivity analysis with the selected prominence held fixed. The selected values are given per fold, cell line, method, and seed in Supplementary Table S7. Separation of the Strong from the Weak reference calls by depth is the area under the receiver operating characteristic curve when the calls on evaluated bins are ranked by the negated profile value at the boundary bin, computed per seed for the predicted profile and once for the measured profile.

Cell-type-specific boundaries are defined from measured Hi-C alone. For a pair of cell lines, a locus qualifies when its measured insulation z-score is below −1.5 in one cell line and above −0.3 in the other, and the contrasting set of the pair pools the loci that qualify in either direction; these thresholds were specified manually. A locus counts as recovered when its predicted z-score is below −1.0 in the cell line with the measured boundary and above −0.5 in the other; the same thresholds are applied to every method. Robustness to the thresholds was assessed on a pre-specified grid in which the two measured thresholds were scaled jointly and the two predicted thresholds were scaled jointly, each by 0.8, 0.9, 1.0, 1.1, or 1.2, giving 25 combinations. All seven comparison methods were scored at every point, and the endpoint and the grid were computed for all six pairs of cell lines. This endpoint is descriptive and complements the aggregate held-out evaluations.

### Scale-resolved correlation and classification controls

Each measured and predicted profile was separated into a smooth component, the symmetric moving average over a half-width of 10, 25, or 50 bins (100, 250, or 500 kb), and a fine-scale component, the profile minus that average. Smoothing runs within contiguous runs of one chromosome only, and one common interior that discards 50 bins at each end of every run is used at all three half-widths, retaining 93.1%–94.9% of the evaluated loci per cell line. Correlations between the measured and predicted components are computed on the decoded scale per seed and averaged over seeds. Between-method differences on the fine-scale component at the 250-kb half-width use a paired block bootstrap over blocks of 200, 500, and 1000 consecutive retained bins (2, 5, and 10 Mb) cut at fold boundaries, with 2000 resamples, the union interval over the three lengths and Benjamini–Hochberg correction of two-sided *P*-values over the 28 tests.

A classification twin of the model was fitted under the same folds, seeds, inputs, and optimisation recipe, differing in target, loss, and the checkpoint-selection metric. Each training bin is labelled positive if it is a local minimum of the fold-standardised measured profile at prominence 0.1; the scalar output is read as a logit under a binary cross-entropy loss whose positive-class weight equals the training negative-to-positive ratio; and the checkpoint with the highest bin-level average precision on the validation chromosome is retained. Its boundaries are maxima of the logit track, called with the procedure above at a prominence selected on the validation chromosome from the 50th, 70th, 80th, 90th, 95th, and 98th percentiles of the validation candidate prominences and then frozen. A second control refits the regression model with the coefficient of the correlation term set to 0. Each control comprises 60 fits and is scored with the procedures of the main analysis.

### Locus illustrations and Micro-C contact ratios

The loci shown in Figs 5 and 7 are illustrative examples from the held-out chromosomes. They are not treated as statistical replicates; quantitative conclusions are based on the aggregate held-out evaluations reported in the corresponding figures and supplementary analyses.

**Figure 7:**
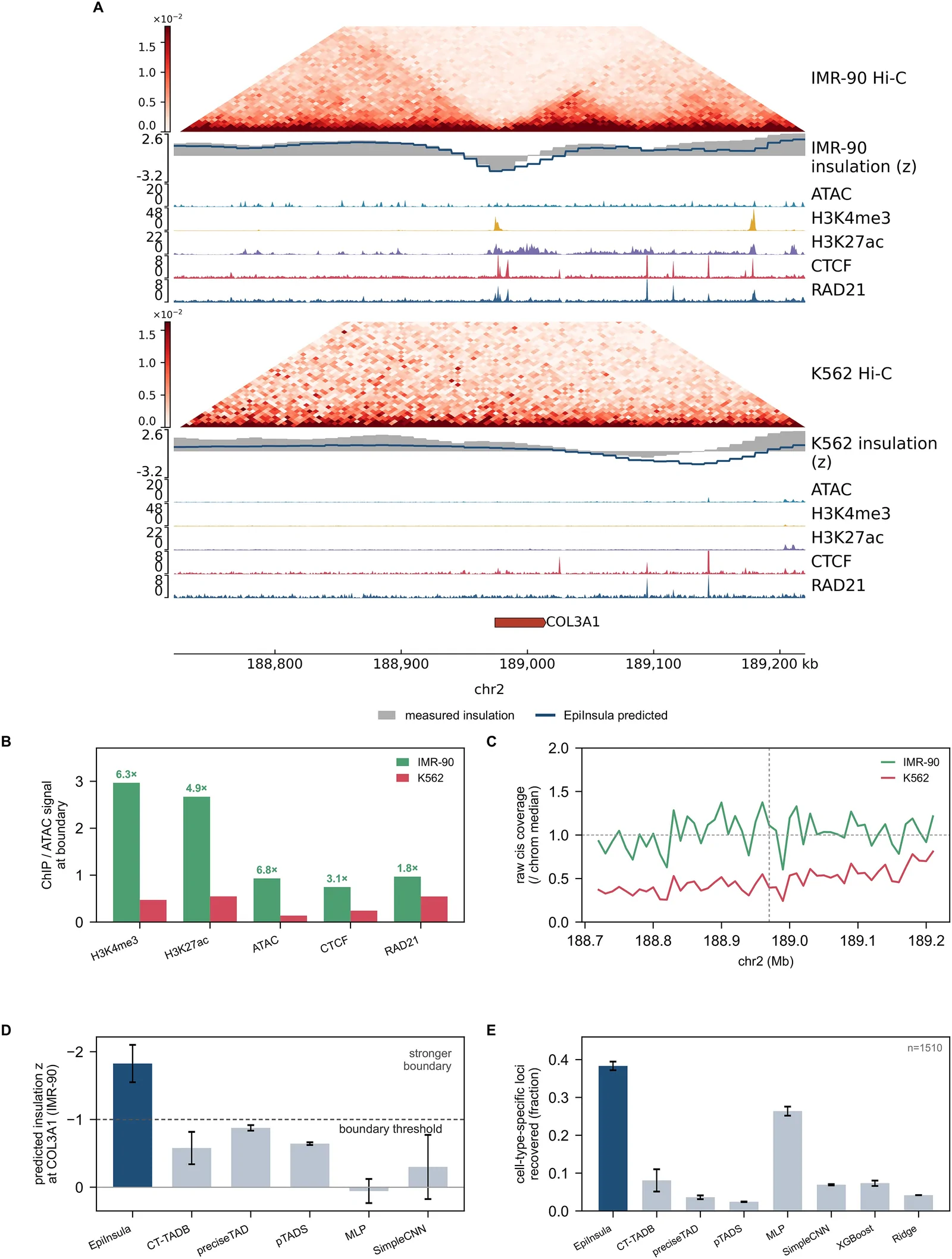
**Cell-type-specific insulation loci in IMR-90 and K562.** The locus shown in panels A and B is an illustrative example from a held-out chromosome and is not treated as a statistical replicate; panel E provides the corresponding aggregate held-out evaluation. All signal tracks shown in panels A and B are model inputs; H3K4me1 is omitted throughout this figure for clarity. (A) Measured Hi-C contact maps, measured and predicted insulation, and five input tracks for IMR-90 and K562 at the illustrative held-out COL3A1 locus, chr2:188.97 Mb. (B) Mean signal within ±5 kb of the boundary for H3K4me3, H3K27ac, ATAC-seq, CTCF, and RAD21 in each cell line; labels give the IMR-90/K562 ratio. These are descriptive values from one window without error bars. (C) Raw cis coverage per 10-kb bin divided by the chromosome median. The vertical dashed line marks the boundary; the horizontal dashed line marks normalised coverage 1. (D) Predicted insulation *z* at COL3A1. The *y*-axis is inverted so stronger insulation appears higher; the dashed line is the predicted-boundary threshold −1.0. Error bars are ± sample standard deviation over *n* = 3 seeds. Ridge regression and the gradient-boosted trees are not shown in panel D. The CT-TADB, preciseTAD, and pTADS results in panels D and E are task-adapted implementations, not the published methods. (E) Fraction of the 1510 contrasting 10-kb loci recovered at the exploratory thresholds defined in Methods, for EpiInsula and all seven comparison methods. Bars are seedwise means and error bars are ± sample standard deviation over *n* = 3 seeds.

GM12878 Micro-C data [50, 51] provide a higher-resolution descriptive comparison and are never used as model input or training target. Contact ratios compare across-centre with within-domain contacts for measured-boundary, measured-flat/model-boundary, and flat-control groups defined on the held-out series. Because one group is defined partly by model output, this is not treated as independent validation. The coverage profiles in Fig. 7C were computed from the released IMR-90 and K562 Hi-C contact matrices [46] and normalised by the chromosome median. Full thresholds and mapping criteria are provided in Supplementary Methods.

### Retrained ablations and inference-time interpretability probes

For each cell line, fold, and seed, the model is retrained after removing CTCF, RAD21, ATAC-seq, or all three histone tracks, producing 240 ablation fits. Each change is measured against the corresponding full-input model.

Seed-42 probes operate on transformed inputs without retraining. Spatial attribution uses mean absolute gradient × input over distance from the window centre. Dose response scales central CTCF or RAD21 signal, and additivity compares their joint response with the sum of single-channel responses. These probes describe fitted-model sensitivity rather than biological causality; implementation details are in Supplementary Methods.

### Cross-cell-type transfer and label-efficiency experiments

For every ordered source–target pair, a source checkpoint is applied without weight updates to the target’s matched held-out chromosomes using the target input scaler. Zero-shot here means that the source weights are applied unchanged and no target label is used. The target inputs are standardised with the target’s own training-chromosome scaler, so this setting uses the target’s unlabelled input statistics and is not strict source-only application. All 16 pairs, five folds, and three seeds are evaluated. Transfer is reported by Pearson correlation; complete pairwise values are given in Supplementary Table S8.

Label-free adaptation recomputes batch-normalisation statistics on unlabelled target training inputs. Labelled adaptation fine-tunes all layers with 0.5%, 1%, 5%, or 25% of target training loci, drawn at random from the fold’s training chromosomes, with early stopping on the fold’s validation chromosome, and compares with random initialisation on the same subset. The source for each target and fold in Fig. 6C and D is the cell line whose checkpoints for that fold give the highest zero-shot correlation on the fold’s validation chromosome, averaged over seeds (Supplementary Table S8, part C); the test chromosomes are read once, after fine-tuning, for the reported value.

## Results

### Held-out prediction accuracy and calibration across cell lines

Across four cell lines, five chromosome-disjoint folds, and three seeds, every evaluated autosomal locus is scored by a model that did not observe its chromosome. Held-out correlation is 0.791 in H1-hESC, 0.780 in GM12878, 0.860 in IMR-90, and 0.774 in K562 (Fig. 2A; Supplementary Table S9), with seedwise standard deviations of at most 0.004.

Across the 21 evaluated autosomes the correlation ranges from 0.708 to 0.835 in H1-hESC, 0.740–0.822 in GM12878, 0.821–0.906 in IMR-90, and 0.661–0.864 in K562 (Fig. 2B), with each chromosome scored by the fold that held it out. Complete chromosome-level values and admitted-locus counts are reported in Fig. 2B and Supplementary Table S2.

The median autosomal correlation is 0.808 in H1-hESC, 0.789 in GM12878, 0.893 in IMR-90 and 0.822 in K562, and the number of autosomes at or above 0.80 is 15, 6, 21 and 17 of 21. IMR-90 reaches this level on all 21 autosomes, whereas GM12878 reaches it on six; the complete chromosome-level distributions are shown in Fig. 2B.

The ordinary-least-squares regression of measured on predicted insulation has slopes of 0.95, 0.95, 0.91, and 0.90, with intercepts within 0.008 of zero (Fig. 2C; Supplementary Table S9). The reverse-regression slopes are 0.66, 0.64, 0.81, and 0.67. These results indicate calibration of the conditional mean together with attenuation of the deepest predicted valleys. Among the deepest 1% of measured insulation values the mean predicted depth is 62.5%, 63.3%, 71.0%, and 52.7% of the mean measured depth in H1-hESC, GM12878, IMR-90, and K562, respectively, so the deepest valleys are recovered only in part. Identity-line coefficients of determination, the reverse-regression slopes, and the depth-recovery values are reported in Supplementary Table S9.

### Matched-protocol comparison ranks the model first on both endpoints

Seven comparison methods were fitted under the contract set out in Methods: the same six tracks, ±700-kb window, five folds, three seeds, and held-out loci. Averaged over the four cell lines, pooled correlation is 0.801 for EpiInsula, 0.703 for CT-TADB, 0.669 for the multilayer perceptron, 0.606 for the plain convolutional network, 0.597 for gradient-boosted trees, 0.588 for pTADS, 0.582 for preciseTAD, and 0.548 for ridge regression (Fig. 3A). EpiInsula ranks first in every cell line; the cell-line-specific margins over the closest comparator are shown in Fig. 3A. The published-method adaptations are task-matched implementations rather than reproductions of the original methods.

All 28 cell-line-by-method correlation differences favour EpiInsula, and every conservative 95% block-bootstrap interval excludes zero (Fig. 3C). Adjusted one-sided probabilities are below 0.001. These comparisons concern task-adapted implementations under the shared contract, not performance reported in the original papers.

Against 4D Nucleome boundary calls, of which there are 9923, 8497, 8059, and 7692 on the evaluated autosomes of the four cell lines, boundary F1 within ±10 kb is highest for EpiInsula in all four cell lines: 0.594, 0.500, 0.597, and 0.540 (Fig. 3B). The closest method, the CT-TADB adaptation, reaches 0.571, 0.457, 0.522, and 0.462, a margin of 0.023, 0.043, 0.075, and 0.078. The remaining six methods reach at most 0.511, by the plain convolutional network in H1-hESC. EpiInsula’s precision is 0.571, 0.523, 0.598 and 0.552 against 0.550, 0.455, 0.534, and 0.481 for the CT-TADB adaptation, and its recall is 0.618, 0.480, 0.596 and 0.530 against 0.594, 0.459, 0.511, and 0.445; both are the highest of the eight methods in every cell line (Fig. 3D). EpiInsula remains first when a match is required at the exact bin or allowed within ±20 kb: it reaches 0.301, 0.234, 0.310 and 0.257 at the exact bin and 0.692, 0.612, 0.699 and 0.648 within ±20 kb, ahead of the second-ranked method by 0.019–0.064 and by 0.025–0.069 (Supplementary Fig. S1). The selected prominence values are given per fold in Supplementary Table S7.

### Insulation scores derived from three retrained contact-map predictors

Three published methods that predict the contact map, Epiphany, C.Origami, and ChromNet, were retrained with their authors’ code and native inputs on our Hi-C matrices under one fold of the partition (test chromosomes 1, 11, 15, 20, and 21), and the insulation score was derived from their predicted maps by the steps of the target definition applied in each method’s native output domain (Methods; Supplementary Table S6). On those chromosomes the derived score correlates with the measured one at 0.533, 0.377, 0.678 and 0.437 for Epiphany, 0.511, 0.346, 0.677 and 0.424 for ChromNet and 0.159, 0.148, 0.280 and 0.289 for C.Origami in H1-hESC, GM12878, IMR-90 and K562, against 0.803, 0.792, 0.877 and 0.821 for EpiInsula on the same fold, seed and chromosomes (Supplementary Fig. S2). The weights released by each group, applied without retraining, give 0.084, 0.418, 0.527 and 0.372 for Epiphany, 0.265, 0.421, 0.683 and 0.542 for ChromNet and 0.178, 0.150, 0.236 and 0.240 for C.Origami; these checkpoints were fitted on chromosome sets that include some of the five test chromosomes, and the cell line they were fitted on coincides with the evaluated one for Epiphany in GM12878 and for the other two methods in IMR-90. The three methods are trained on the contact map and scored here on a quantity derived from it; the values measure the support their output gives to the insulation endpoint.

### The lead is retained on the fine-scale component of the profile

Separating each profile into a smooth component and a fine-scale component at a 250-kb half-width (Methods) places EpiInsula’s correlation on the fine-scale component at 0.798, 0.774, 0.861 and 0.801 in H1-hESC, GM12878, IMR-90 and K562. These values are 0.007 above, 0.006 below, 0.001 above, and 0.027 above the pooled correlation of the same cell line. At the 100-kb half-width the fine-scale correlation is 0.804, 0.778, 0.855 and 0.807, and at 500 kb it is 0.797, 0.771, 0.860, and 0.794 (Supplementary Fig. S3; Supplementary Table S10). CT-TADB is the closest method on this component, at 0.745, 0.732, 0.811 and 0.707 at 250 kb, and all 28 cell-line-by-method differences on the fine-scale component are positive with union block-bootstrap intervals excluding zero, the smallest being 0.042 [0.038, 0.046] for CT-TADB in GM12878; every corrected two-sided *P* is below .001.

### A classification twin and the correlation-term control

A classification twin of the network, fitted on binarised boundary labels under the same folds, seeds and optimisation recipe (Methods), reaches a boundary F1 within ±10 kb of 0.506, 0.404, 0.508, and 0.471 in H1-hESC, GM12878, IMR-90, and K562, against 0.594, 0.500, 0.597, and 0.540 for the regression model, and an average precision over ranked boundary candidates of 0.492, 0.371, 0.496, and 0.463 against 0.596, 0.504, 0.614, and 0.537 (Supplementary Table S11). Its logit track correlates with the measured insulation score at −0.357, −0.397, −0.469, and −0.416, the sign following from boundaries being minima. Refitting the regression model without the correlation term of the loss changes none of these quantities by more than 0.004 in any cell line, with overlapping per-seed ranges (Supplementary Table S11), and leaves the lead of the regression target over the classification target intact.

### Predicted depth orders reference boundaries by strength

Among the 4D Nucleome reference calls that fall on evaluated bins, the predicted depth at the boundary bin separates the Strong from the Weak calls with an area under the receiver operating characteristic curve of 0.750, 0.743, 0.699, and 0.690 in H1-hESC, GM12878, IMR-90 and K562, against 0.897, 0.859, 0.793, and 0.794 for the measured depth (Supplementary Table S12).

### Input ablation reveals cell-line-specific dependence and graded perturbation response

Removing an input group and refitting from random initialisation lowers held-out accuracy in 15 of the 16 cell-line-by-condition comparisons whose unadjusted 200-bin block-bootstrap intervals exclude zero (Fig. 4B; Supplementary Table S13). The largest changes involve removal of the three histone tracks together and removal of RAD21 in H1-hESC, whereas the CTCF change in IMR-90 is near zero. Fold-level spread for every cell-line-by-condition comparison is shown in Supplementary Fig. S4, and descriptive summaries relating removal effects to the dose response are given in Supplementary Fig. S5. Effects vary across cell lines and are conditional on the inputs retained in each model; they should not be interpreted as causal biological contributions.

The fitted-model probes show that attribution is concentrated near the window centre and that predicted insulation changes gradually when central CTCF or RAD21 signal is scaled (Fig. 4A and C). The joint response varies across cell lines (Fig. 4D). These manipulations operate on transformed model inputs and report sensitivity of the fitted function; they do not establish chromatin mechanisms.

### Locus-level profiles illustrate agreement in boundary position

At the locus scale the predicted profile follows the measured one in position while remaining closer to zero at the valleys, the same behaviour the calibration slopes and depth-recovery figures quantify genome-wide (Fig. 5A and B). The window shown in Fig. 5B covers the *BCL6* locus in GM12878, chr3:187.60–187.88 Mb, where the measured profile reaches its local minimum at 187.73 Mb and the predicted profile reaches its own minimum at 187.72 Mb, one bin away. A Micro-C map of the same cell line at higher resolution resolves the same boundary. The displayed loci are illustrative examples and are not treated as statistical replicates; the corresponding quantitative conclusions are based on aggregate held-out evaluations. The boundary at the *BCL6* position carries co-localised CTCF and RAD21 occupancy; both are model input channels, so their agreement with the predicted boundary describes what the model reads rather than establishing that the boundary is real.

The median across-boundary/within-domain Micro-C contact ratio is 0.084 for measured boundaries, 0.120 for measured-flat/model-boundary loci and 0.142 for flat controls. Because the middle group is defined partly by model output and Micro-C assays the same cell type, this comparison is descriptive rather than independent validation.

### Fine-tuning enables cross-cell-line transfer at low label cost

Applied to a cell line on which it was not fitted, with no weight update, a model reaches a mean correlation of 0.561 across the 12 ordered cross-cell pairs, compared with a within-cell mean of 0.801 (Fig. 6A; Supplementary Table S8). The complete directional matrix and seedwise dispersion are reported in Supplementary Table S8.

Label-free recomputation of batch-normalisation statistics on target-cell training inputs changes the mean cross-cell correlation from 0.561 to 0.594 (Fig. 6B). This is a modest descriptive improvement without weight updates or target labels; target-level values are shown in Fig. 6B.

Transfer performance varies by source and target cell line; the full zero-shot matrix and target-specific label-free changes are provided in Fig. 6A and B, and Supplementary Table S8.

The source selected on each fold’s validation chromosome is GM12878 in 12 of the 20 target-by-fold combinations, H1-hESC and K562 in three each and IMR-90 in two (Supplementary Table S8, part C). With 1% of target training loci, fine-tuning from the selected source reaches 0.737, 0.750, 0.840, and 0.750 across the four targets, or 93.2%–97.7% of within-cell correlation (Fig. 6C). Models trained from random initialisation on the same subsets reach 0.596, 0.556, 0.721, and 0.619 (Fig. 6D); at an equal label budget the source weights add 0.141, 0.194, 0.119, and 0.131. The complete label-budget curves are shown in Fig. 6C and D.

### Predicted profiles recover boundaries differing between IMR-90 and K562

The genome-wide endpoint is defined from measured contact maps alone. At the displayed IMR-90/K562 thresholds, 1510 of 242 041 jointly scored bins show a measured strong-versus-flat contrast between the two cell lines. On this fixed set, and with the same predicted thresholds applied to every method, EpiInsula recovers 0.383 of the contrasts, with per-seed values of 0.371, 0.385, and 0.393, compared with 0.264 for the multilayer perceptron and 0.024–0.081 for the remaining six methods, of which ridge regression and the gradient-boosted trees recover 0.042 and 0.073 (Fig. 7E). Over the 25 combinations of the pre-specified threshold grid, in which the contrasting set contains 890–2961 loci, EpiInsula’s recovered fraction is 0.281–0.425; it ranks first at every point, the multilayer perceptron ranks second at every point, and the margin between the two is 0.063–0.147 (Supplementary Fig. S6; Supplementary Table S14). On the other five cell-line pairs the same endpoint gives contrasting sets of 1145–2017 loci at the unscaled thresholds and recovered fractions of 0.343 to 0.435 for EpiInsula. EpiInsula ranks first at all 125 grid points of those pairs, with the multilayer perceptron second at 120 and the plain convolutional network at five; over the 150 points of the six pairs the margin over the second-ranked method is 0.036–0.224 (Supplementary Table S15). This descriptive analysis at the displayed thresholds is distinct from the within-cell boundary comparison in Fig. 3B.

At *COL3A1*, chr2:188.97 Mb, an IMR-90-specific boundary is present and is absent in K562 (Fig. 7A). The chromatin at that position carries IMR-90-specific signal in every input channel, strongest for ATAC at 6.8-fold, H3K4me3 at 6.3-fold and H3K27ac at 4.9-fold (Fig. 7B); these are model input channels, so their co-localisation with the predicted boundary describes what the model reads. Coverage across the interval is flat and at the chromosome median in IMR-90, the cell line in which the boundary is called, while K562 shows a regional coverage depletion at the same position (Fig. 7C), so the called boundary does not coincide with a coverage anomaly in the cell line that carries it. The predicted z-score is −1.83 with a spread of 0.28 over seeds for EpiInsula, against −0.88, −0.64, −0.58, −0.30, and +0.06 for preciseTAD, pTADS, CT-TADB, the plain convolutional network and the multilayer perceptron (Fig. 7D). This locus is shown for illustration and is not treated as a statistical replicate; the preceding aggregate analysis provides the quantitative context.

## Discussion

One recurring formulation of computational chromatin-architecture prediction is boundary classification, which gives relatively limited attention to the insulation profile itself [24, 35, 36]. The present study instead predicts the continuous insulation score across four human cell lines under chromosome-disjoint evaluation. Held-out correlations range from 0.774 to 0.860, and every evaluated locus is scored by a model that did not observe its chromosome. The measured-on-predicted regression slopes of 0.90–0.95 indicate calibration of the conditional mean, while the reverse regressions show attenuation of individual amplitudes. The output can therefore be analysed as a continuous profile, with that amplitude limitation kept explicit. The network is assembled from established components and no architectural novelty is claimed; the contribution is the target, the evaluation design and the characterisation of the fitted model.

Seven comparison methods were evaluated under the same inputs, window, folds, seeds and held-out loci. Under this contract, EpiInsula ranks first in every cell line, and all block-bootstrap intervals for paired correlation differences exclude zero. Because three published approaches were adapted to continuous regression, these results compare task-matched implementations rather than reproducing the original methods. Three published contact-map predictors, retrained on the same Hi-C matrices with their own inputs and code, yield derived insulation scores that correlate with the measured profile at 0.15–0.68 on chromosomes on which EpiInsula reaches 0.79–0.88; those methods are trained on the contact map and their output is converted to the insulation score afterwards, so the comparison measures the support each output gives to this endpoint. Within the same backbone, the classification twin, which differs in target, loss and checkpoint selection, gives the lower boundary F1 and average precision, and the regression model keeps that lead without the correlation term of its loss. The scale decomposition shows that the correlation is not carried by the smooth component of the profile alone.

The ranking of methods depends on the endpoint. At the displayed exploratory IMR-90/K562 thresholds, EpiInsula recovers 0.383 of measured strong-versus-flat contrasts, compared with 0.264 for the multilayer perceptron and 0.081 for the strongest of the other six methods, and EpiInsula ranks first at all 25 points of the threshold grid and at every grid point of each of the other five cell-line pairs. This result supports evaluating cell-type-specific contrasts alongside pooled correlation. It is a descriptive aggregate comparison and does not establish a biological mechanism at any individual locus.

Input-group ablations show that predictive dependence on the six tracks varies across cell lines. Removing the histone group produces the largest change in three cell lines and removing RAD21 the largest in the remaining cell line; the CTCF change in IMR-90 is near zero when the other inputs are retained. Because the input tracks are correlated, each ablation is conditional on the remaining channels. The predictive weight carried by RAD21 is consistent with the long-established association of cohesin with CTCF at sites of transcriptional insulation [52, 53], although the present analysis measures predictive dependence rather than that association itself. These analyses describe predictive information and fitted-model sensitivity, not regulatory mechanism.

The depth and shape of the profile carry information that a boundary label does not. Boundaries stratified by insulation strength differ in CTCF occupancy, and super-enhancers are preferentially insulated by strong boundaries [54]; boundary width, which correlates with the distribution of CTCF sites and with chromatin features, modulates insulation between neighbouring domains [55]; and deletion of a single boundary CTCF motif permits ectopic enhancer contacts, with consequences for gene expression and development [56]. In the predictions reported here, predicted depth separates the Strong from the Weak calls of the reference set with an area under the curve below that of the measured depth (Results, ‘Predicted depth orders reference boundaries by strength’), so the predicted profile carries a graded record of boundary strength, less sharply ordered than the measured profile, that a binary call does not. Within a cell line for which the model has been fitted, such a profile can locate boundaries on chromosomes without a contact map and order them by predicted strength, and the profiles of two cell lines can be compared locus by locus to nominate positions at which the domain landscape differs between them. Extension to a cell type without a contact map depends on transfer, whose zero-shot and fine-tuned correlations are reported in the Results. Because the model reads the epigenomic tracks directly, it can also indicate how a change in those tracks propagates to the predicted profile, as the dose–response probes illustrate. These uses generate hypotheses for contact-map and perturbation experiments rather than replace them.

Cross-cell experiments show that zero-shot performance is lower than within-cell performance, while recomputing batch-normalisation statistics provides a modest label-free improvement. With 1% of target-cell training loci, fine-tuning reaches 93.2%–97.7% of within-cell correlation with the source selected on each fold’s validation chromosome. These findings support fine-tuning when target labels are available but do not establish label-free generalisation to untested cell types.

Three limitations define the scope of the study. EpiInsula requires six cell-type-specific epigenomic tracks, which may not be available in primary or clinical samples. Its fixed ±700-kb context may miss longer-range structure. Finally, the attribution, input-scaling, and cell-type-specific analyses are computational and descriptive; they do not establish regulatory causality.

## Conclusion

The formulation of chromatin architecture prediction as calibrated regression on a continuous profile provides a framework for investigating genome organisation across cellular contexts. Experimental technologies continue to generate contact maps at increasing resolution and across diverse biological conditions, and sequence-based models now predict multiple regulatory modalities within a single framework [57]. Methods that predict the insulation profile itself should be evaluated where cell types differ as well as where they agree, because the ranking of methods can change between the two settings.

**Key Points**

- EpiInsula regresses continuous 10-kb Hi-C insulation from six epigenomic tracks under chromosome-disjoint evaluation.
- Held-out correlations are 0.774–0.860, with conditional-mean calibration slopes of 0.90–0.95 across four cell lines.
- Evaluated against seven alternative models with the same inputs and chromosome partitions, EpiInsula gives the highest correlation and, with thresholds selected on validation chromosomes, the highest boundary F1 in every cell line; the lead is retained on the fine-scale component of the profile, and three retrained contact-map predictors give lower correlation on the derived insulation score.
- At the displayed IMR-90/K562 thresholds, EpiInsula recovers 0.383 of contrasting 10-kb loci and ranks first against all seven comparison methods at every point of a 25-point threshold grid and in all six cell-line pairs; this descriptive endpoint complements pooled correlation.
- With the source selected on each fold’s validation chromosome, 1% target labels recover 93.2%–97.7% of within-cell correlation.

## Supplementary Material

Supplementary_material_bbag589

## Data Availability

The data underlying this article are publicly available. Twenty of the twenty-four epigenomic input tracks are from the ENCODE portal [42] and four, for H1-hESC, from the 4D Nucleome portal [45]; the insulation-score targets and the Hi-C contact matrices from which they and the Fig. 7C coverage were derived are from the 4D Nucleome portal [46]; the reference boundary calls are from the 4D Nucleome portal [49]; and the GM12878 Micro-C contact matrix used for descriptive comparison and display, which is not a model input, is from the Gene Expression Omnibus under accession GSE225201 (GSE225201_GM12878_Micro-C.nodups.mapq_20.mcool) [51]. Per-file accessions, assemblies and per-file notes for every cell line are listed in Supplementary Table S1. A project repository is listed at https://github.com/Yin-Shen/EpiInsula. The manuscript and supplementary information define the methods and analyses reported in the submitted version.
