# Supplementary material for: EpiInsula: calibrated regression of the Hi-C insulation score from cell-type-specific epigenomic signal
Source: Brief Bioinform. 2026 Oct 26;27(5):bbag589. doi: 10.1093/bib/bbag589 (PMC13704792; doi:10.1093/bib/bbag589)
Supplement: Supplementary file 1 [file supplementary_material_bbag589.docx]

**Supplementary Information**

*EpiInsula: calibrated regression of the Hi-C insulation score from cell-type-specific epigenomic signal*

# Supplementary Methods

## Data acquisition and file-level checks

The model uses four human cell lines on GRCh38: H1-hESC, GM12878, IMR-90 and K562. Inputs are six cell-type-specific signal tracks: ATAC-seq, CCCTC-binding factor (CTCF), H3K4me1, H3K4me3, H3K27ac and the cohesin subunit RAD21. All were stored as processed fold-change-over-control bigWig files. Twenty of the 24 inputs are ENCODE files and four are 4D Nucleome files. Supplementary Table S1 lists each accession, repository, assembly and file-specific note. The listed records were released on GRCh38 when accessed. Local files were checked for readability and chromosome metadata.

Only the six epigenomic channels enter the model. Sequence-derived inputs were not used.

## Insulation target and locus construction

The target is the diamond insulation score. For a genomic bin *i* and half-window *w*, the numerator is the mean balanced contact frequency in the diamond connecting the *w* upstream bins to the *w* downstream bins. This value is divided by the median over the mappable region of the same chromosome arm and transformed on a base-2 logarithmic scale. Values near zero indicate typical across-bin contact for the arm; increasingly negative values indicate stronger local insulation. Released 4D Nucleome insulation tracks were generated with cooltools 0.2.0 from GRCh38 contact matrices at 5-kb resolution with a 100-kb insulation window and were aggregated to the non-overlapping 10-kb prediction grid. Each 10-kb bin receives the mean of its finite base-resolution target values.

A locus comprises one 10-kb target bin and its stored epigenomic context. It was admitted if at least half of the target values within the bin were finite, the stored ±1-Mb window lay inside the chromosome, and every input track had finite coverage over at least half of that window. Remaining non-finite signal values in admitted windows were replaced by zero before scaling. The filter admits 254,834 H1-hESC, 258,012 GM12878, 256,850 IMR-90 and 258,635 K562 autosomal loci (1,028,331 in total). Supplementary Table S2 reports chromosome-level retention.

## Input transformation and architecture

Signals were binned at 1 kb; the model reads the central ±700 kb, producing a 6 × 1,400 input matrix. Half-width selection is described in Materials and methods and Supplementary Table S3. Within each cell line and fold, every channel was centred on the training-chromosome median and divided by the training-chromosome interquartile range. The estimates used at most two million values per channel, allocated to chromosomes in proportion to admitted loci with deterministic within-chromosome sampling. Scaled values underwent an inverse hyperbolic sine transform and clipping at ±30. Targets were standardised from training chromosomes only and decoded before regression metrics were computed. Boundary analyses used the standardised profile.

EpiInsula is a one-dimensional dilated residual convolutional network. A width-7 stem maps six inputs to 128 channels, followed by batch normalisation and a Gaussian error linear unit. Eight residual blocks have width-3 convolutions at dilations 1, 2, 4, 8, 16, 32, 64 and 128. Each block includes batch normalisation, the dilated convolution, a Gaussian error linear unit, a pointwise convolution, dropout of 0.3 and an identity skip. No pooling or striding is used. The read-out concatenates the 128 central-position features and the 128-channel mean over all 1,400 positions; a two-layer perceptron with 192 hidden units predicts one score. The model has 583,681 trainable parameters. The central path has a 517-bin (±258-kb) receptive field; positions outside it can contribute through the global mean. Supplementary Table S4 gives the complete settings and parameter decomposition.

## Optimisation and chromosome-disjoint evaluation

The training objective is smooth L1 loss on the standardised target plus 0.3 times one minus the within-batch Pearson correlation. Models used AdamW with a learning rate of 0.0003, weight decay of 0.001 and batch size 256. Training included two warm-up epochs, cosine decay over a 60-epoch budget, gradient-norm clipping at 1.0, bfloat16 mixed precision and joint reversal of all six tracks with probability 0.5. Early stopping used validation correlation with patience 12 and retained the epoch with the highest validation correlation. Across the 60 primary fits, training stopped after a median of 18 epochs. One NVIDIA RTX 5090 required a median of 15 min per fit (15.2 GPU-hours in total).

Whole chromosomes define every partition. Folds 1 and 2 tested chromosomes {1, 11, 15, 20, 21} and {2, 9, 14, 19, 22}, respectively. Folds 3–5 tested {3, 8, 10, 17}, {4, 7, 12, 18} and {5, 6, 13, 16}. Their validation chromosomes were 14, 15, 16, 13 and 17, respectively. The remaining 16 or 17 autosomes trained each fold. A fold’s scaler, target standardisation, weights and early-stopping decision use only its training and validation chromosomes. Seeds 42, 13 and 99 determine initialisation, batch order, reversal draws and label subsets. Concatenating the five held-out blocks gives one prediction per admitted autosomal locus per seed. Chromosome 8 is excluded from every held-out evaluation (Materials and methods).

## Metrics and uncertainty

The primary metric is Pearson correlation on the decoded held-out series. Additional quantities are the ordinary-least-squares slope and intercept of measured on predicted insulation, the reverse slope of predicted on measured insulation, and the coefficient of determination against the identity line. A forward slope near one means that the conditional mean of measured insulation at a given prediction is close to that prediction; it does not imply uncompressed individual amplitudes. Depth recovery is the mean predicted value divided by the mean measured value among the lowest 1% of measured scores. Except for explicitly labelled seed-42 inference probes and the correlation after averaging predictions across seeds, every reported estimate is calculated per seed and then averaged. Dispersion over seeds is the sample standard deviation (denominator *n* − 1; *n* = 3), except for the population standard deviation displayed in Figure 6C.

Genomic block-bootstrap intervals preserve chromosome identity. Within each chromosome, ordered loci were cut at coordinate gaps and divided into blocks of 50, 100 or 200 contiguous 10-kb bins. Two thousand chromosome-stratified resamples were drawn. The ablation analysis reports the 2.5th–97.5th percentiles from 200-bin blocks without multiplicity correction. For method comparisons, the interval is the union across the three block lengths; the one-sided probability is the largest across lengths and is adjusted over 28 tests spanning four cell lines and seven comparators by the Benjamini–Hochberg procedure. The smallest resolved probability is 1/2,000. These intervals are conditional on the observed chromosome set; fold-level spread is shown separately in Supplementary Figure S4.

## Comparison implementations

The comparison models are fitted under the contract stated in Materials and methods. CT-TADB was adapted for the continuous insulation target (Materials and methods; Supplementary Table S5). The preciseTAD adaptation replaces boundary classification with random-forest regression. The pTADS adaptation blends random-forest and LASSO predictions (Supplementary Table S5).

We also evaluated a multilayer perceptron (labelled MLP in the figures), a plain convolutional network (SimpleCNN), ridge regression (Ridge) and gradient-boosted trees (XGBoost); the two networks are specified in Supplementary Table S5. Ridge regression, gradient-boosted trees and the random-forest and LASSO components of the preciseTAD and pTADS comparisons use 36 summaries: per-channel mean, maximum and standard deviation over the window, the central-bin value, and means within central ±25-kb and ±100-kb windows. Ridge regression and gradient-boosted trees use the full training split; the preciseTAD and pTADS models, including the LASSO component, use the training subsample given in Supplementary Table S5.

## Contact-map prediction methods

Epiphany, C.Origami and ChromNet were run with the authors’ released code. Retraining used fold 1 of the chromosome partition (training chromosomes 2–10, 12, 13, 16–19 and 22; validation chromosome 14; test chromosomes 1, 11, 15, 20 and 21), one fit per cell line at seed 42. Epiphany: inputs DNase-seq, H3K27ac, H3K27me3, H3K4me3 and CTCF at 100-bp resolution (Supplementary Table S1); target the HiC-DC+ normalised 1-Mb diagonal band at 10-kb resolution built from the same contact matrices as the insulation targets; model, dataset, loss with its released adversarial weighting (λ = 0.95), optimiser (Adam, learning rate 0.0001, weight decay 0.0005, halving every 16 epochs), batch size 1 and 55-epoch budget as released; the training loop was re-implemented to apply the chromosome split, score the full validation set each epoch, retain the validation-optimal checkpoint, apply the seed and read the per-cell-line data directories, and early stopping at patience 12 on the validation loss was added. C.Origami and ChromNet: inputs DNA sequence (GRCh38), CTCF and ATAC-seq (CTCF supplied as log2(fold change + 1) to C.Origami, which applies no internal transform, and as fold change to ChromNet, which applies log(x + 1) internally); target the balanced 2,097,152-bp contact map at 8,192-bp resolution built from the same matrices; Adam with learning rate 0.0002, 10 warm-up epochs with cosine decay, gradient clipping at 1, 80 epochs, validation-optimal checkpoint retained, per-device batch size 8 on one GPU (the released configuration uses four GPUs); patches applied the chromosome split and library-version compatibility, and the number of data-loading workers was reduced. Published weights: Epiphany 10-kb GM12878 model (Zenodo record 7795868); C.Origami IMR-90 model (Zenodo record 7226561); ChromNet checkpoint released with the paper. The insulation score of a predicted map is the diamond mean of linear-domain contacts at a half-width of 100 kb (10 bins on the 10-kb grid; 12 bins of 8,192 bp for C.Origami and ChromNet), averaged over overlapping windows, log2-transformed and resampled to the 10-kb grid. From each chromosome arm the log2 median over its mappable bins is subtracted (arms from the hg38 centromere annotation; mappable bins are those with a finite measured score). Pearson correlation with the measured insulation score is pooled over the five test chromosomes.

## Scale decomposition and classification controls

The scale decomposition follows Materials and methods; contiguous runs of 101 or fewer 10-kb bins were dropped before trimming. The classification twin and the loss ablation are defined in Materials and methods (‘Scale-Resolved Correlation and Classification Controls’); both use the five folds and three seeds.

## Boundary endpoints

The released calls are 5-kb BED intervals; their midpoints are assigned to the 10-kb grid. Prominence selection, boundary calling and matching follow Materials and methods; Supplementary Table S7 reports the selected values per fold and the F1 at the three tolerances.

The cell-type-specific endpoint, its thresholds and the robustness grid are defined in Materials and methods. All eight methods were scored at every grid point, with ridge-regression and gradient-boosted-tree predictions converted to the standardised scale with each fold’s training-set target standardiser; Supplementary Table S14 lists every value for IMR-90/K562 and Supplementary Table S15 the endpoint and grid summary for all six cell-line pairs. This endpoint is a descriptive analysis at the displayed thresholds rather than a prospective threshold-selection procedure.

## Locus displays and Micro-C comparison

The loci shown in Figures 5 and 7 are illustrative examples from the held-out chromosomes. They are not treated as statistical replicates; quantitative conclusions are based on the aggregate held-out evaluations reported in the corresponding figures and supplementary analyses. The GM12878 Micro-C matrix associated with GSE225201 was used only for this comparison and was not a model input or training target. At 2-kb resolution, the descriptive contact ratio within ±120 kb is the mean across-centre rectangle divided by the mean of the two within-domain triangles. Loci with more than 15% unmappable bins were excluded. The groups were measured boundaries (measured *z* < −1.5), measured-flat/model-boundary loci (measured *z* > −0.4 and predicted *z* < −1.3) and flat controls (both scores > −0.4). Because the model-output-defined group and the Micro-C data refer to the same cell type, this analysis is interpreted as a descriptive same-cell-type comparison rather than independent validation. Figure 7C used the released GRCh38 10-kb multi-resolution cooler matrices 4DNFIJTOIGOI (IMR-90) and 4DNFI18UHVRO (K562); raw cis coverage per bin was normalised by the median across the corresponding chromosome.

## Ablation, response probes and transfer

For each cell line, fold and seed, separate models were retrained after removing CTCF, RAD21, ATAC-seq or all three histone tracks, giving 240 fits. The reported change is relative to the full six-track model. Seed-42 inference probes use transformed inputs and do not retrain the model. Spatial attribution is mean absolute gradient × input, aggregated in 25-kb distance bins on held-out loci and normalised within each fold to sum to one. Dose response multiplies the central ±20 kb of CTCF or RAD21 by 0, 0.5, 1.5 or 2.0; because transformed tracks are median-centred, factor zero sets the slice to the training median. The interaction residual is the joint attenuation response minus the sum of the two single-channel responses. Figure 4D shows fold means and dispersion across five folds at seed 42. These probes describe fitted-model sensitivity rather than biological causality.

For transfer, the source checkpoint for cell line A, fold *f* and seed *s* is applied without weight updates to cell line B’s fold-*f* held-out chromosomes. The target’s own input scaler is used. All 16 ordered pairs are evaluated; the four diagonal cells reproduce the within-cell result through the transfer path. Label-free adaptation resets and re-accumulates the nine batch-normalisation layers’ running statistics on unlabelled target training-chromosome inputs. Labelled adaptation fine-tunes all layers using 0.5%, 1%, 5% or 25% of target training loci, drawn at random from the fold’s training chromosomes with a seed-determined subset, with early stopping on the fold’s validation chromosome, and compares with random-initialised training on the same subset. The source for each target and fold is the cell line with the highest zero-shot correlation on that fold’s validation chromosome (Supplementary Table S8, part C); the test chromosomes are read once, after fine-tuning.

# Supplementary Table S1

Supplementary Table S1. Data provenance and accession numbers. Repository, accession, assembly and notes for all six epigenomic input tracks in each cell line, four insulation targets, four reference-boundary files, the GM12878 Micro-C matrix, the two Hi-C matrices used for Figure 7C coverage, and the DNase-seq and H3K27me3 tracks and the genome sequence read only by the retrained contact-map predictors. The listed records were released on GRCh38 when accessed. Local files were checked for readability and chromosome compatibility.

| **Category** | **Cell line** | **Assay / feature** | **Repository** | **Accession** | **Assembly** | **Note** |
| --- | --- | --- | --- | --- | --- | --- |
| Model input epigenomic tracks (six per cell line) | H1-hESC | ATAC-seq | 4DN | 4DNFICPNO4M5 | GRCh38 |  |
| Model input epigenomic tracks (six per cell line) | H1-hESC | CTCF ChIP-seq | ENCODE | ENCFF038RVZ | GRCh38 |  |
| Model input epigenomic tracks (six per cell line) | H1-hESC | H3K4me1 ChIP-seq | 4DN | 4DNFIDPEPJ5B | GRCh38 |  |
| Model input epigenomic tracks (six per cell line) | H1-hESC | H3K4me3 ChIP-seq | 4DN | 4DNFIQP1BBB3 | GRCh38 |  |
| Model input epigenomic tracks (six per cell line) | H1-hESC | H3K27ac ChIP-seq | 4DN | 4DNFIRN137C9 | GRCh38 |  |
| Model input epigenomic tracks (six per cell line) | H1-hESC | RAD21 ChIP-seq | ENCODE | ENCFF791XXJ | GRCh38 |  |
| Model input epigenomic tracks (six per cell line) | GM12878 | ATAC-seq | ENCODE | ENCFF603BJO | GRCh38 |  |
| Model input epigenomic tracks (six per cell line) | GM12878 | CTCF ChIP-seq | ENCODE | ENCFF734CUT | GRCh38 |  |
| Model input epigenomic tracks (six per cell line) | GM12878 | H3K4me1 ChIP-seq | ENCODE | ENCFF564KBE | GRCh38 |  |
| Model input epigenomic tracks (six per cell line) | GM12878 | H3K4me3 ChIP-seq | ENCODE | ENCFF287HAO | GRCh38 |  |
| Model input epigenomic tracks (six per cell line) | GM12878 | H3K27ac ChIP-seq | ENCODE | ENCFF469WVA | GRCh38 |  |
| Model input epigenomic tracks (six per cell line) | GM12878 | RAD21 ChIP-seq | ENCODE | ENCFF571ZJJ | GRCh38 |  |
| Model input epigenomic tracks (six per cell line) | IMR-90 | ATAC-seq | ENCODE | ENCFF282RNO | GRCh38 | † |
| Model input epigenomic tracks (six per cell line) | IMR-90 | CTCF ChIP-seq | ENCODE | ENCFF105FHL | GRCh38 |  |
| Model input epigenomic tracks (six per cell line) | IMR-90 | H3K4me1 ChIP-seq | ENCODE | ENCFF221MJG | GRCh38 |  |
| Model input epigenomic tracks (six per cell line) | IMR-90 | H3K4me3 ChIP-seq | ENCODE | ENCFF376ZIM | GRCh38 |  |
| Model input epigenomic tracks (six per cell line) | IMR-90 | H3K27ac ChIP-seq | ENCODE | ENCFF699OAR | GRCh38 |  |
| Model input epigenomic tracks (six per cell line) | IMR-90 | RAD21 ChIP-seq | ENCODE | ENCFF048PZI | GRCh38 |  |
| Model input epigenomic tracks (six per cell line) | K562 | ATAC-seq | ENCODE | ENCFF754EAC | GRCh38 |  |
| Model input epigenomic tracks (six per cell line) | K562 | CTCF ChIP-seq | ENCODE | ENCFF979PWH | GRCh38 |  |
| Model input epigenomic tracks (six per cell line) | K562 | H3K4me1 ChIP-seq | ENCODE | ENCFF607SUJ | GRCh38 |  |
| Model input epigenomic tracks (six per cell line) | K562 | H3K4me3 ChIP-seq | ENCODE | ENCFF253TOF | GRCh38 |  |
| Model input epigenomic tracks (six per cell line) | K562 | H3K27ac ChIP-seq | ENCODE | ENCFF381NDD | GRCh38 |  |
| Model input epigenomic tracks (six per cell line) | K562 | RAD21 ChIP-seq | ENCODE | ENCFF320RTQ | GRCh38 |  |
| Insulation-score training target (4D Nucleome Hi-C) | H1-hESC | insulation score (bigWig) | 4DN | 4DNFIUK3UVZX | GRCh38 | ‡ |
| Insulation-score training target (4D Nucleome Hi-C) | GM12878 | insulation score (bigWig) | 4DN | 4DNFIUNXO8CU | GRCh38 | ‡ |
| Insulation-score training target (4D Nucleome Hi-C) | IMR-90 | insulation score (bigWig) | 4DN | 4DNFIZFI8U3R | GRCh38 | ‡ |
| Insulation-score training target (4D Nucleome Hi-C) | K562 | insulation score (bigWig) | 4DN | 4DNFIXU7QLG6 | GRCh38 | ‡ |
| Reference boundary calls (same workflow run as the target) | H1-hESC | boundary calls (bed) | 4DN | 4DNFIEKE967M | GRCh38 | ¶ |
| Reference boundary calls (same workflow run as the target) | GM12878 | boundary calls (bed) | 4DN | 4DNFII786ZNB | GRCh38 | ¶ |
| Reference boundary calls (same workflow run as the target) | IMR-90 | boundary calls (bed) | 4DN | 4DNFIMNT2VYL | GRCh38 | ¶ |
| Reference boundary calls (same workflow run as the target) | K562 | boundary calls (bed) | 4DN | 4DNFI4EFYN3Q | GRCh38 | ¶ |
| Higher-resolution map used for the illustrative locus analysis and display | GM12878 | Micro-C contact matrix (mcool) | GEO | GSE225201 (GSE225201_GM12878_Micro-C.nodups.mapq_20.mcool) | GRCh38 | § |
| Source contact matrix used for Figure 7C coverage | IMR-90 | in situ Hi-C contact matrix (mcool) | 4DN | 4DNFIJTOIGOI | GRCh38 | chromosome-median-normalised raw cis coverage in Figure 7C |
| Source contact matrix used for Figure 7C coverage | K562 | in situ Hi-C contact matrix (mcool) | 4DN | 4DNFI18UHVRO | GRCh38 | chromosome-median-normalised raw cis coverage in Figure 7C |
| Inputs read only by the retrained contact-map predictors | H1-hESC | DNase-seq | ENCODE | ENCFF232GUZ | GRCh38 | read-depth normalised signal; Epiphany |
| Inputs read only by the retrained contact-map predictors | GM12878 | DNase-seq | ENCODE | ENCFF960FMM | GRCh38 | read-depth normalised signal; Epiphany |
| Inputs read only by the retrained contact-map predictors | IMR-90 | DNase-seq | ENCODE | ENCFF971HXR | GRCh38 | read-depth normalised signal; Epiphany |
| Inputs read only by the retrained contact-map predictors | K562 | DNase-seq | ENCODE | ENCFF414OGC | GRCh38 | read-depth normalised signal; Epiphany |
| Inputs read only by the retrained contact-map predictors | H1-hESC | H3K27me3 ChIP-seq | ENCODE | ENCFF345VHG | GRCh38 | fold change over control; Epiphany |
| Inputs read only by the retrained contact-map predictors | GM12878 | H3K27me3 ChIP-seq | ENCODE | ENCFF919DOR | GRCh38 | fold change over control; Epiphany |
| Inputs read only by the retrained contact-map predictors | IMR-90 | H3K27me3 ChIP-seq | ENCODE | ENCFF366BVS | GRCh38 | fold change over control; Epiphany |
| Inputs read only by the retrained contact-map predictors | K562 | H3K27me3 ChIP-seq | ENCODE | ENCFF242ENK | GRCh38 | fold change over control; Epiphany |
| Inputs read only by the retrained contact-map predictors | — | DNA sequence | — | — | GRCh38 | primary assembly; C.Origami and ChromNet |

† Nuclear-fraction preparation (ENCSR200OML), not whole-cell; retained and flagged.

‡ Source contact matrix: H1-hESC 4DNFI82R42AD, GM12878 4DNFIXVAKX9Q, IMR-90 4DNFIJTOIGOI, K562 4DNFI18UHVRO.

¶ Reference for boundary-level performance; Strong and Weak calls both retained.

§ Illustrative locus analysis and display only; never a model input and never a training target.

# Supplementary Table S2

Supplementary Table S2. Per-chromosome locus retention under the quality filter. Candidate 10-kb bins, admitted loci and retention percentage are listed for every cell line. The autosomal total is 1,028,331 loci; chromosome 8 is not evaluated (Materials and methods).

| **Chromosome** | **Candidate 10-kb bins** | **H1-hESC admitted** | **H1-hESC retention (%)** | **GM12878 admitted** | **GM12878 retention (%)** | **IMR-90 admitted** | **IMR-90 retention (%)** | **K562 admitted** | **K562 retention (%)** |
| --- | --- | --- | --- | --- | --- | --- | --- | --- | --- |
| 1 | 24,895 | 21,326 | 85.66 | 21,620 | 86.84 | 21,476 | 86.27 | 21,808 | 87.60 |
| 2 | 24,219 | 22,735 | 93.87 | 23,068 | 95.25 | 22,922 | 94.64 | 23,269 | 96.08 |
| 3 | 19,829 | 19,228 | 96.97 | 19,260 | 97.13 | 19,275 | 97.21 | 19,287 | 97.27 |
| 4 | 19,021 | 18,421 | 96.85 | 18,456 | 97.03 | 18,454 | 97.02 | 18,474 | 97.12 |
| 5 | 18,153 | 17,040 | 93.87 | 17,275 | 95.16 | 17,202 | 94.76 | 17,349 | 95.57 |
| 6 | 17,080 | 16,344 | 95.69 | 16,550 | 96.90 | 16,498 | 96.59 | 16,580 | 97.07 |
| 7 | 15,934 | 14,573 | 91.46 | 14,888 | 93.44 | 14,745 | 92.54 | 14,979 | 94.01 |
| 8 | 14,513 | 13,756 | 94.78 | 13,778 | 94.94 | 13,749 | 94.74 | 13,879 | 95.63 |
| 9 | 13,839 | 10,636 | 76.86 | 10,723 | 77.48 | 10,672 | 77.12 | 9,860 | 71.25 |
| 10 | 13,379 | 12,390 | 92.61 | 12,604 | 94.21 | 12,527 | 93.63 | 12,755 | 95.34 |
| 11 | 13,508 | 12,478 | 92.37 | 12,637 | 93.55 | 12,614 | 93.38 | 12,736 | 94.28 |
| 12 | 13,327 | 12,695 | 95.26 | 12,722 | 95.46 | 12,739 | 95.59 | 12,789 | 95.96 |
| 13 | 11,436 | 9,296 | 81.29 | 9,388 | 82.09 | 9,341 | 81.68 | 9,423 | 82.40 |
| 14 | 10,704 | 8,558 | 79.95 | 8,601 | 80.35 | 8,597 | 80.32 | 8,628 | 80.61 |
| 15 | 10,199 | 7,394 | 72.50 | 7,515 | 73.68 | 7,417 | 72.72 | 7,542 | 73.95 |
| 16 | 9,033 | 6,763 | 74.87 | 7,059 | 78.15 | 6,937 | 76.80 | 7,161 | 79.28 |
| 17 | 8,325 | 7,124 | 85.57 | 7,364 | 88.46 | 7,312 | 87.83 | 7,430 | 89.25 |
| 18 | 8,037 | 7,130 | 88.71 | 7,220 | 89.83 | 7,167 | 89.18 | 7,253 | 90.25 |
| 19 | 5,861 | 5,095 | 86.93 | 5,253 | 89.63 | 5,206 | 88.82 | 5,291 | 90.27 |
| 20 | 6,444 | 5,683 | 88.19 | 5,745 | 89.15 | 5,728 | 88.89 | 5,788 | 89.82 |
| 21 | 4,670 | 3,047 | 65.25 | 3,132 | 67.07 | 3,104 | 66.47 | 3,137 | 67.17 |
| 22 | 5,081 | 3,122 | 61.44 | 3,154 | 62.07 | 3,168 | 62.35 | 3,217 | 63.31 |
| X | 15,604 | 13,677 | 87.65 | 14,123 | 90.51 | 13,988 | 89.64 | 14,280 | 91.51 |
| Autosomes (22) | 287,487 | 254,834 | 88.64 | 258,012 | 89.75 | 256,850 | 89.34 | 258,635 | 89.96 |

# Supplementary Table S3

Supplementary Table S3. Input-window sweep on the development partition. Validation Pearson correlation (seedwise mean ± SD over seeds 42, 13 and 99); training chromosomes 1–7, 9, 10, 12–15, 17–21 and X, validation chromosome 8, input scalers fitted per channel on 2,000,000 values sampled every 1,000 bp in chromosome order from the training chromosomes. The retained half-width is the smallest whose mean over cell lines lies within 0.001 of the highest mean (Materials and methods); chromosome 8 is excluded from every held-out evaluation.

| **Input half-width (kb)** | **H1-hESC** | **GM12878** | **IMR-90** | **K562** | **Mean over cell lines** |
| --- | --- | --- | --- | --- | --- |
| ±100 | 0.773 ± 0.002 | 0.772 ± 0.004 | 0.858 ± 0.002 | 0.767 ± 0.003 | 0.793 |
| ±250 | 0.819 ± 0.002 | 0.812 ± 0.003 | 0.894 ± 0.001 | 0.808 ± 0.003 | 0.833 |
| ±400 | 0.828 ± 0.002 | 0.818 ± 0.002 | 0.898 ± 0.003 | 0.821 ± 0.005 | 0.841 |
| ±550 | 0.831 ± 0.001 | 0.823 ± 0.002 | 0.901 ± 0.001 | 0.827 ± 0.009 | 0.845 |
| ±700 | 0.833 ± 0.001 | 0.825 ± 0.004 | 0.903 ± 0.003 | 0.830 ± 0.003 | 0.848 |
| ±850 | 0.832 ± 0.003 | 0.824 ± 0.002 | 0.903 ± 0.002 | 0.833 ± 0.005 | 0.848 |
| ±1,000 | 0.833 ± 0.002 | 0.825 ± 0.003 | 0.902 ± 0.001 | 0.834 ± 0.004 | 0.848 |

# Supplementary Table S4

Supplementary Table S4. Model architecture, training and inputs. Settings required to reinstantiate the six-channel model, including the parameter decomposition summing to 583,681 trainable parameters.

## A. Model architecture

| **Component** | **Setting** | **Notes** |
| --- | --- | --- |
| Model family | 1D dilated residual CNN | no pooling or strided convolution at any depth |
| Input channels | 6 epigenomic tracks | ATAC-seq, CTCF, H3K4me1, H3K4me3, H3K27ac, RAD21 |
| Stem | kernel 7, 128 channels | batch normalisation and GELU follow |
| Residual blocks | 8 dilated blocks | dilations 1, 2, 4, 8, 16, 32, 64, 128 |
| Block design | pre-activation residual block | batch norm, dilated conv, GELU, pointwise conv, dropout |
| Skip path | unmodified identity | gradients reach the stem without a normalisation layer |
| Padding | equal to the dilation | sequence length preserved at every depth |
| Kernel sizes | stem 7, blocks 3 | odd widths keep the length |
| Dropout | 0.3 | in every block and in the read-out head |
| Read-out pooling | centre bin and global mean | concatenated into a 256-dimensional vector |
| Read-out head | two layers, 192 hidden units | one scalar per locus |
| Receptive field | 517 bins (±258 kb) | 37% of the window; the rest enters via the global mean |
| Cell-identity label | none | a separate model is fitted for each cell line |
| Trainable parameters | 583,681 | 90% of them in the residual tower |
| stem | 5,760 | convolution 5,504 and batch norm 256 |
| residual tower | 528,384 | 8 × 66,048, independent of dilation |
| read-out head | 49,537 | hidden layer 49,344 and output 193 |

*Supplementary Table S4 (continued)*

## B. Training

| **Component** | **Setting** | **Notes** |
| --- | --- | --- |
| Optimiser | AdamW |  |
| Learning rate | 0.0003 | two-epoch warm-up, then cosine decay |
| Weight decay | 0.001 |  |
| Batch size | 256 |  |
| Maximum epochs | 60 | never reached; median run 18 epochs |
| Early stopping | patience 12 | criterion: Pearson r on the validation chromosome |
| Loss | smooth L1 (β = 1) + 0.3 (1 − r) | r is the Pearson correlation within the mini-batch |
| Gradient clipping | max-norm 1.0 |  |
| Mixed precision | bfloat16 |  |
| Augmentation | genomic-axis reversal, p = 0.5 | per mini-batch, all six tracks; target unchanged |
| Seeds | 42, 13, 99 | the seedwise mean is the primary estimand |
| Hardware | one NVIDIA RTX 5090 | median 15 min per model, 15.2 GPU-hours in total |
| Interpreter | Python 3.13.12 |  |
| Deep-learning framework | PyTorch 2.11.0 (CUDA 13.0) |  |
| Comparison-method libraries | scikit-learn 1.8.0, XGBoost 3.2.0 | ridge, random-forest and gradient-boosted arms |
| Numerical and I/O libraries | NumPy 2.4.4, SciPy 1.17.1, h5py 3.16.0, pyBigWig 0.3.25 |  |

## C. Inputs and preprocessing

| **Component** | **Setting** | **Notes** |
| --- | --- | --- |
| Input window | ±700 kb | 1,400 bins at 1-kb resolution |
| Input tracks | 6 bigWig signal channels | one set per cell line |
| Prediction target | 10-kb insulation score | at the central bin of the window |
| Signal scaling | robust scaler | median and IQR per channel, training chromosomes only |
| Compression | inverse hyperbolic sine | stabilises the heavy right tail of read density |
| Clipping | ±30 | after compression; a numerical guard, not outlier control |
| Storage precision | float32 HDF5 |  |
| Target standardisation | z-score | mean and SD from the training chromosomes |

# Supplementary Table S5

Supplementary Table S5. Comparison-method architectures, fixed settings, search spaces and selected values. The four networks use the common recipe of Supplementary Table S4; their only data-determined value is the stopping epoch, selected by validation Pearson. Selected values are counted over the 60 fold-by-cell-line-by-seed fits (ridge regression: 20 fold-by-cell-line fits, one per fold and cell line); epochs and boosting rounds are counted from 1.

| **Method** | **Method group** | **Architecture or learner** | **Trainable parameters** | **Fixed settings** | **Search space and selection rule** | **Selected values** |
| --- | --- | --- | --- | --- | --- | --- |
| EpiInsula | This work | Dilated residual convolutional network (Supplementary Table S4) | 583,681 | Common recipe (Supplementary Table S4) | — | Best epoch median 6; training stopped after a median of 18 epochs |
| CT-TADB | Published method, task-adapted | Convolutional–Transformer hybrid (published architecture). Convolutional stem: Conv1d 6→64 (k = 9), 2× max-pooling, Conv1d 64→64 (k = 9), 2× max-pooling, 2× max-pooling (172 tokens). Six broadcast tokens carrying the per-track window means; learned positional embedding over the 178 tokens (11,392 parameters). Residual-free 4-head self-attention block (dim 64) with layer normalisation and feed-forward 64→32→64. Linear read-out 11,392→1. Adaptation: the six tracks replace the one-hot DNA input of the convolutional branch, their per-track window means replace the published scalar epigenomic features of the broadcast branch, and the linear insulation-score output replaces the sigmoid boundary output | 84,321 | Common recipe; dropout 0.3 after each convolution, 0.1 in the attention block | — | Best epoch median 15; training stopped after a median of 27 epochs |
| MLP | Architecture control | Average-pooling to 32 bins per channel (192 inputs); fully connected layers 192→494→494→494→1 with Gaussian error linear units | 584,897 | Common recipe; dropout 0.3 after each hidden layer | — | Best epoch median 20; training stopped after a median of 32 epochs |
| SimpleCNN | Architecture control | Four Conv1d layers 6→96→96→96→96 (k = 7, no dilation) with batch normalisation and Gaussian error linear units; centre + global-mean read-out 192→192→1 with a Gaussian error linear unit | 235,969 | Common recipe; dropout 0.3 after the second to fourth convolutions and in the read-out | — | Best epoch median 12; training stopped after a median of 24 epochs |
| preciseTAD | Published method, task-adapted | Random-forest regressor on the 36 window-summary features | — | scikit-learn RandomForestRegressor, 400 trees; fitted on at most 120,000 training loci per fold | (max_depth, max_features, min_samples_leaf) ∈ {(none, sqrt, 5), (none, 0.5, 5), (none, sqrt, 20), (24, sqrt, 5)}, selected by validation Pearson | (none, 0.5, 5) in 39; (none, sqrt, 5) in 13; (24, sqrt, 5) in 8 |
| pTADS | Published method, task-adapted | The preciseTAD random forest (the same fitted model per fold, cell line and seed) plus a LASSO linear model on the 36 features, blended | — | LASSO fitted on the preciseTAD training subsample | LASSO alpha ∈ {0.0001, 0.0003, 0.001, 0.003, 0.01, 0.03} and blend weight on the random forest ∈ {0, 0.1, …, 1.0}, selected by validation Pearson | alpha 0.0001 in 27, 0.001 in 18, 0.003 in 6, 0.0003 in 6, 0.01 in 3, 0.03 in 0; blend weight 0.6 in 21, 0.7 in 18, 0.5 in 11, 0.4 in 4, 0.8 in 3, 0.9 in 3 |
| XGBoost | Feature baseline | Gradient-boosted trees on the 36 features | — | max_depth 6, eta 0.1, subsample 0.8, colsample_bytree 0.8, hist; objective squared error; up to 500 rounds with early stopping (30 rounds) on validation root-mean-square error | None beyond the stopping round | Stopping round retained for the 20 seed-42 fits (10 to 265 rounds, median 167); not retained for seeds 13 and 99 |
| Ridge | Feature baseline | Ridge regression on the 36 features (closed form) | — | — | alpha ∈ {0.1, 1, 10, 100}, selected by validation Pearson | alpha 100 in 14, 0.1 in 6 |

# Supplementary Table S6

Supplementary Table S6. Relationship between EpiInsula and three published contact-map prediction methods. Inputs, predicted object, output resolution, training target and training chromosomes are given for each method, with the Pearson correlation between the insulation score derived from its predicted map and the measured insulation score over test chromosomes 1, 11, 15, 20 and 21 (fold 1), both after retraining on our data with the authors’ code and native inputs (one fit per cell line, seed 42) and with the weights released by each group. EpiInsula is given on the same fold, seed and chromosomes. The last column records which of the five test chromosomes were in the training set of the released weights.

| **Method** | **Inputs** | **Predicted object** | **Output resolution** | **Training target** | **Training chromosomes** | **Derived-insulation Pearson r, retrained on our data (H1-hESC / GM12878 / IMR-90 / K562)** | **Derived-insulation Pearson r, released weights** | **Overlap of the released weights with the five test chromosomes** |
| --- | --- | --- | --- | --- | --- | --- | --- | --- |
| EpiInsula (this work) | ATAC-seq, CTCF, H3K4me1, H3K4me3, H3K27ac, RAD21 (1-kb bins, ±700 kb) | 10-kb insulation score (direct) | 10 kb | Insulation score from the released 4D Nucleome track | Fold 1 of the five-fold partition (16 autosomes; validation chr14) | 0.803 / 0.792 / 0.877 / 0.821 | — | — |
| Epiphany [25] | DNase-seq, H3K27ac, H3K27me3, H3K4me3, CTCF (100-bp bins) | 1-Mb diagonal contact band | 10 kb | HiC-DC+ normalised contacts from the same matrices | As above (retrained); released GM12878 model trained on all autosomes except chr3, chr11 and chr17 | 0.533 / 0.377 / 0.678 / 0.437 | 0.084 / 0.418 / 0.527 / 0.372 | Released model: chr1, chr15, chr20 and chr21 were in its training set; trained on GM12878 |
| C.Origami [26] | DNA sequence, CTCF, ATAC-seq | 2.1-Mb contact map | 8,192 bp | Balanced contacts from the same matrices (log(x + 1) domain) | As above (retrained); released IMR-90 model trained on all autosomes except chr10 and chr15 | 0.159 / 0.148 / 0.280 / 0.289 | 0.178 / 0.150 / 0.236 / 0.240 | Released model: chr1, chr11, chr20 and chr21 were in its training set; trained on IMR-90 |
| ChromNet [27] | DNA sequence, CTCF, ATAC-seq | 2.1-Mb contact map (multi-task, cell-type head) | 8,192 bp | Balanced contacts from the same matrices (log(x + 1) domain) | As above (retrained); released model trained on chr1–chr16 (IMR-90 Hi-C) | 0.511 / 0.346 / 0.677 / 0.424 | 0.265 / 0.421 / 0.683 / 0.542 | Released model: chr1, chr11 and chr15 were in its training set; trained on IMR-90 |

# Supplementary Table S7

Supplementary Table S7. Boundary F1, precision and recall with validation-selected prominence. For each cell line and method, columns give the prominence selected on each fold’s validation chromosome (folds 1–5) for seeds 42, 13 and 99, the seedwise-mean F1 with its SD at three matching tolerances, precision and recall within ±10 kb and the number of predicted boundaries (seedwise means), against the 4D Nucleome reference calls (Materials and methods). No selected value lies at either end of the grid. Ridge regression has no stochastic component, so its three seeds coincide. On the six-value grid of the submitted version (0.05 to 0.4), the same validation-selected protocol gives F1 within ±10 kb of 0.594, 0.498, 0.596 and 0.534 for EpiInsula, 0.571, 0.457, 0.522 and 0.462 for the CT-TADB adaptation and at most 0.452 for the remaining six methods.

| **Cell line** | **Method** | **Method group** | **Prominence per fold (seed 42)** | **Prominence per fold (seed 13)** | **Prominence per fold (seed 99)** | **F1 exact bin** | **SD** | **F1 ±10 kb** | **SD** | **F1 ±20 kb** | **SD** | **Precision ±10 kb (SD)** | **Recall ±10 kb (SD)** | **Predicted boundaries** | **Reference boundaries** |
| --- | --- | --- | --- | --- | --- | --- | --- | --- | --- | --- | --- | --- | --- | --- | --- |
| H1-hESC | EpiInsula | This work | 0.5/0.4/0.5/0.8/0.5 | 0.5/0.4/0.4/0.6/0.6 | 0.5/0.3/0.3/0.5/0.4 | 0.301 | 0.0081 | 0.594 | 0.0026 | 0.692 | 0.0009 | 0.571 (0.0142) | 0.618 (0.0207) | 10,742 | 9,923 |
| H1-hESC | CT-TADB | Published method, task-adapted | 0.1/0.15/0.1/0.3/0.2 | 0.2/0.15/0.1/0.3/0.15 | 0.2/0.1/0.1/0.2/0.2 | 0.282 | 0.0057 | 0.571 | 0.0102 | 0.667 | 0.0062 | 0.550 (0.0075) | 0.594 (0.0147) | 10,729 | 9,923 |
| H1-hESC | preciseTAD | Published method, task-adapted | 0.4/0.6/0.5/0.6/0.4 | 0.4/0.6/0.3/0.5/0.4 | 0.5/0.6/0.4/0.5/0.3 | 0.143 | 0.0013 | 0.379 | 0.0009 | 0.546 | 0.0022 | 0.346 (0.0052) | 0.420 (0.0092) | 12,039 | 9,923 |
| H1-hESC | pTADS | Published method, task-adapted | 0.4/0.8/0.5/0.6/0.4 | 0.5/0.5/0.4/0.6/0.5 | 0.5/0.6/0.5/0.5/0.5 | 0.149 | 0.0029 | 0.394 | 0.0049 | 0.563 | 0.0069 | 0.369 (0.0049) | 0.424 (0.0066) | 11,412 | 9,923 |
| H1-hESC | MLP | Architecture control | 0.15/0.4/0.5/0.4/0.6 | 0.6/0.5/0.4/0.6/0.8 | 0.2/0.3/0.5/0.4/1.0 | 0.093 | 0.0035 | 0.280 | 0.0049 | 0.450 | 0.0076 | 0.258 (0.0238) | 0.309 (0.0222) | 11,994 | 9,923 |
| H1-hESC | SimpleCNN | Architecture control | 0.8/1.0/1.0/0.8/1.0 | 0.8/1.0/1.0/0.8/1.2 | 1.0/1.0/1.0/1.0/0.8 | 0.260 | 0.0044 | 0.511 | 0.0039 | 0.586 | 0.0033 | 0.457 (0.0026) | 0.580 (0.0141) | 12,610 | 9,923 |
| H1-hESC | XGBoost | Feature baseline | 0.5/0.8/0.8/0.6/0.5 | 0.8/0.8/0.8/0.6/0.4 | 0.6/0.8/0.8/0.6/0.6 | 0.160 | 0.0023 | 0.401 | 0.0030 | 0.557 | 0.0053 | 0.371 (0.0076) | 0.436 (0.0121) | 11,692 | 9,923 |
| H1-hESC | Ridge | Feature baseline | 0.6/0.6/0.5/0.8/0.3 | 0.6/0.6/0.5/0.8/0.3 | 0.6/0.6/0.5/0.8/0.3 | 0.136 | 0.0000 | 0.368 | 0.0000 | 0.530 | 0.0000 | 0.320 (0.0000) | 0.433 (0.0000) | 13,417 | 9,923 |
| GM12878 | EpiInsula | This work | 0.6/0.6/0.8/0.4/0.6 | 0.6/0.5/0.6/0.6/0.5 | 0.5/0.6/0.8/0.5/1.0 | 0.234 | 0.0060 | 0.500 | 0.0045 | 0.612 | 0.0027 | 0.523 (0.0089) | 0.480 (0.0103) | 7,804 | 8,497 |
| GM12878 | CT-TADB | Published method, task-adapted | 0.15/0.3/0.15/0.3/0.2 | 0.15/0.2/0.15/0.15/0.1 | 0.15/0.3/0.3/0.3/0.3 | 0.198 | 0.0138 | 0.457 | 0.0208 | 0.579 | 0.0168 | 0.455 (0.0238) | 0.459 (0.0199) | 8,573 | 8,497 |
| GM12878 | preciseTAD | Published method, task-adapted | 0.3/0.4/0.3/0.3/0.4 | 0.2/0.4/0.3/0.4/0.4 | 0.3/0.5/0.5/0.5/0.4 | 0.107 | 0.0004 | 0.289 | 0.0017 | 0.422 | 0.0028 | 0.261 (0.0135) | 0.325 (0.0229) | 10,621 | 8,497 |
| GM12878 | pTADS | Published method, task-adapted | 0.3/0.5/0.3/0.3/0.4 | 0.2/0.4/0.3/0.5/0.5 | 0.3/0.5/0.3/0.5/0.4 | 0.105 | 0.0011 | 0.288 | 0.0013 | 0.422 | 0.0014 | 0.264 (0.0055) | 0.316 (0.0083) | 10,188 | 8,497 |
| GM12878 | MLP | Architecture control | 0.5/0.5/0.6/0.8/0.5 | 0.5/0.5/0.6/0.6/0.5 | 0.2/0.6/0.3/0.4/0.3 | 0.103 | 0.0049 | 0.280 | 0.0050 | 0.413 | 0.0069 | 0.286 (0.0339) | 0.279 (0.0268) | 8,431 | 8,497 |
| GM12878 | SimpleCNN | Architecture control | 0.8/1.0/0.8/0.8/1.0 | 0.8/0.8/1.0/0.8/1.0 | 0.8/0.8/1.0/0.8/0.6 | 0.189 | 0.0042 | 0.403 | 0.0045 | 0.491 | 0.0025 | 0.364 (0.0092) | 0.450 (0.0086) | 10,519 | 8,497 |
| GM12878 | XGBoost | Feature baseline | 0.5/0.5/0.5/0.3/0.6 | 0.4/0.4/0.8/0.6/0.5 | 0.4/0.5/0.4/0.6/0.3 | 0.123 | 0.0009 | 0.305 | 0.0017 | 0.431 | 0.0040 | 0.266 (0.0081) | 0.358 (0.0121) | 11,466 | 8,497 |
| GM12878 | Ridge | Feature baseline | 0.3/0.5/0.5/0.3/0.3 | 0.3/0.5/0.5/0.3/0.3 | 0.3/0.5/0.5/0.3/0.3 | 0.086 | 0.0000 | 0.237 | 0.0000 | 0.367 | 0.0000 | 0.193 (0.0000) | 0.305 (0.0000) | 13,413 | 8,497 |
| IMR-90 | EpiInsula | This work | 0.6/0.5/0.5/0.5/0.5 | 0.5/0.3/0.3/0.6/0.4 | 0.4/0.4/0.4/0.5/0.4 | 0.310 | 0.0063 | 0.597 | 0.0055 | 0.699 | 0.0036 | 0.598 (0.0205) | 0.596 (0.0093) | 8,039 | 8,059 |
| IMR-90 | CT-TADB | Published method, task-adapted | 0.2/0.15/0.1/0.2/0.15 | 0.2/0.15/0.1/0.2/0.2 | 0.15/0.1/0.15/0.2/0.2 | 0.246 | 0.0124 | 0.522 | 0.0148 | 0.640 | 0.0102 | 0.534 (0.0075) | 0.511 (0.0216) | 7,717 | 8,059 |
| IMR-90 | preciseTAD | Published method, task-adapted | 0.4/0.4/0.4/0.5/0.4 | 0.4/0.5/0.3/0.5/0.5 | 0.4/0.5/0.4/0.4/0.4 | 0.125 | 0.0020 | 0.334 | 0.0002 | 0.489 | 0.0017 | 0.316 (0.0022) | 0.355 (0.0026) | 9,054 | 8,059 |
| IMR-90 | pTADS | Published method, task-adapted | 0.5/0.5/0.4/0.4/0.4 | 0.3/0.5/0.5/0.3/0.3 | 0.3/0.4/0.4/0.3/0.3 | 0.127 | 0.0019 | 0.344 | 0.0060 | 0.510 | 0.0044 | 0.314 (0.0240) | 0.383 (0.0196) | 9,871 | 8,059 |
| IMR-90 | MLP | Architecture control | 0.6/0.6/0.5/1.0/0.6 | 0.6/0.5/0.5/0.8/0.6 | 0.6/0.4/0.6/0.3/0.8 | 0.130 | 0.0018 | 0.340 | 0.0043 | 0.483 | 0.0031 | 0.372 (0.0203) | 0.315 (0.0129) | 6,833 | 8,059 |
| IMR-90 | SimpleCNN | Architecture control | 0.5/0.8/0.8/0.8/0.6 | 0.8/1.0/0.6/0.8/0.8 | 0.8/1.0/1.0/0.8/0.8 | 0.226 | 0.0074 | 0.461 | 0.0094 | 0.540 | 0.0080 | 0.411 (0.0320) | 0.529 (0.0291) | 10,450 | 8,059 |
| IMR-90 | XGBoost | Feature baseline | 0.3/0.4/0.8/0.6/0.6 | 0.5/0.6/0.8/0.8/0.6 | 0.4/0.6/0.8/0.6/0.5 | 0.139 | 0.0029 | 0.349 | 0.0051 | 0.509 | 0.0092 | 0.323 (0.0234) | 0.383 (0.0220) | 9,622 | 8,059 |
| IMR-90 | Ridge | Feature baseline | 0.6/0.6/0.6/0.6/0.5 | 0.6/0.6/0.6/0.6/0.5 | 0.6/0.6/0.6/0.6/0.5 | 0.114 | 0.0000 | 0.306 | 0.0000 | 0.464 | 0.0000 | 0.298 (0.0000) | 0.314 (0.0000) | 8,495 | 8,059 |
| K562 | EpiInsula | This work | 0.5/0.5/0.4/0.6/0.5 | 0.8/0.6/0.6/0.6/0.8 | 0.6/0.8/0.5/0.5/0.5 | 0.257 | 0.0028 | 0.540 | 0.0038 | 0.648 | 0.0024 | 0.552 (0.0220) | 0.530 (0.0142) | 7,399 | 7,692 |
| K562 | CT-TADB | Published method, task-adapted | 0.2/0.2/0.1/0.15/0.15 | 0.2/0.1/0.1/0.2/0.1 | 0.3/0.2/0.15/0.3/0.1 | 0.200 | 0.0034 | 0.462 | 0.0120 | 0.579 | 0.0100 | 0.481 (0.0165) | 0.445 (0.0124) | 7,122 | 7,692 |
| K562 | preciseTAD | Published method, task-adapted | 0.4/0.5/0.2/0.2/0.2 | 0.3/0.4/0.2/0.3/0.2 | 0.4/0.5/0.2/0.2/0.2 | 0.120 | 0.0030 | 0.325 | 0.0030 | 0.462 | 0.0046 | 0.290 (0.0083) | 0.369 (0.0070) | 9,812 | 7,692 |
| K562 | pTADS | Published method, task-adapted | 0.3/0.6/0.4/0.4/0.3 | 0.3/0.6/0.4/0.4/0.4 | 0.4/0.5/0.3/0.5/0.3 | 0.121 | 0.0014 | 0.330 | 0.0006 | 0.474 | 0.0021 | 0.313 (0.0053) | 0.349 (0.0052) | 8,596 | 7,692 |
| K562 | MLP | Architecture control | 0.4/0.1/0.5/0.4/0.6 | 0.3/0.15/0.4/0.1/0.6 | 0.3/0.1/0.6/0.15/0.5 | 0.087 | 0.0012 | 0.244 | 0.0028 | 0.382 | 0.0069 | 0.203 (0.0114) | 0.306 (0.0172) | 11,628 | 7,692 |
| K562 | SimpleCNN | Architecture control | 1.2/1.0/0.8/1.0/0.8 | 1.0/0.8/0.8/0.6/1.0 | 1.2/0.8/1.0/1.0/1.0 | 0.216 | 0.0026 | 0.457 | 0.0013 | 0.544 | 0.0024 | 0.436 (0.0196) | 0.481 (0.0270) | 8,515 | 7,692 |
| K562 | XGBoost | Feature baseline | 0.4/0.6/0.8/0.4/0.15 | 0.5/0.6/0.8/0.6/0.15 | 0.5/0.6/0.8/0.4/0.2 | 0.127 | 0.0017 | 0.339 | 0.0021 | 0.477 | 0.0014 | 0.313 (0.0063) | 0.371 (0.0124) | 9,113 | 7,692 |
| K562 | Ridge | Feature baseline | 0.6/0.6/0.6/0.6/0.5 | 0.6/0.6/0.6/0.6/0.5 | 0.6/0.6/0.6/0.6/0.5 | 0.107 | 0.0000 | 0.304 | 0.0000 | 0.445 | 0.0000 | 0.283 (0.0000) | 0.327 (0.0000) | 8,893 | 7,692 |

Reference boundaries by label class: H1-hESC 9,923 = 6,330 Strong + 3,593 Weak; GM12878 8,497 = 4,570 Strong + 3,927 Weak; IMR-90 8,059 = 5,233 Strong + 2,826 Weak; K562 7,692 = 4,180 Strong + 3,512 Weak.

# Supplementary Table S8

Supplementary Table S8. Pairwise cross-cell-type transfer. Part A gives all 16 ordered source–target pairs per seed on the test chromosomes. Diagonal entries are within-cell controls. Part B gives the mean over three source cell lines before and after label-free batch-normalisation adaptation. Part C gives, for each target and fold, the seedwise-mean zero-shot correlation of every candidate source on the fold’s validation chromosome and the source selected for Figure 6C–D; selection used the unrounded means. Changes in part B are computed before rounding.

## A. Ordered cell-line pairs

| **Source cell line** | **Target cell line** | **Regime** | **r, seed 42** | **r, seed 13** | **r, seed 99** | **r (seedwise mean)** | **SD over seeds** |
| --- | --- | --- | --- | --- | --- | --- | --- |
| H1-hESC | H1-hESC | Within cell line, recomputed through the transfer path | 0.792 | 0.790 | 0.791 | 0.791 | 0.0014 |
| H1-hESC | GM12878 | Zero-shot, no weight update | 0.570 | 0.572 | 0.582 | 0.575 | 0.0069 |
| H1-hESC | IMR-90 | Zero-shot, no weight update | 0.732 | 0.729 | 0.754 | 0.738 | 0.0140 |
| H1-hESC | K562 | Zero-shot, no weight update | 0.455 | 0.433 | 0.449 | 0.446 | 0.0111 |
| GM12878 | H1-hESC | Zero-shot, no weight update | 0.582 | 0.583 | 0.578 | 0.581 | 0.0024 |
| GM12878 | GM12878 | Within cell line, recomputed through the transfer path | 0.780 | 0.780 | 0.780 | 0.780 | 0.0001 |
| GM12878 | IMR-90 | Zero-shot, no weight update | 0.782 | 0.783 | 0.776 | 0.780 | 0.0042 |
| GM12878 | K562 | Zero-shot, no weight update | 0.563 | 0.539 | 0.578 | 0.560 | 0.0200 |
| IMR-90 | H1-hESC | Zero-shot, no weight update | 0.363 | 0.332 | 0.331 | 0.342 | 0.0181 |
| IMR-90 | GM12878 | Zero-shot, no weight update | 0.601 | 0.604 | 0.603 | 0.603 | 0.0018 |
| IMR-90 | IMR-90 | Within cell line, recomputed through the transfer path | 0.856 | 0.860 | 0.862 | 0.860 | 0.0030 |
| IMR-90 | K562 | Zero-shot, no weight update | 0.218 | 0.162 | 0.203 | 0.194 | 0.0293 |
| K562 | H1-hESC | Zero-shot, no weight update | 0.549 | 0.542 | 0.545 | 0.545 | 0.0036 |
| K562 | GM12878 | Zero-shot, no weight update | 0.654 | 0.663 | 0.653 | 0.657 | 0.0057 |
| K562 | IMR-90 | Zero-shot, no weight update | 0.702 | 0.728 | 0.703 | 0.711 | 0.0148 |
| K562 | K562 | Within cell line, recomputed through the transfer path | 0.772 | 0.779 | 0.771 | 0.774 | 0.0041 |

## B. Label-free adaptation on the target cell line

| **Target cell line** | **Zero-shot r, mean over the three source cell lines** | **r after label-free adaptation** | **Change on adaptation** | **Within-cell r for the same target** |
| --- | --- | --- | --- | --- |
| H1-hESC | 0.489 | 0.523 | +0.034 | 0.791 |
| GM12878 | 0.611 | 0.636 | +0.024 | 0.780 |
| IMR-90 | 0.743 | 0.736 | −0.007 | 0.860 |
| K562 | 0.400 | 0.483 | +0.083 | 0.774 |

*Supplementary Table S8 (continued)*

## C. Zero-shot correlation on each fold’s validation chromosome (source selection for Figure 6C–D)

| **Target cell line** | **Fold** | **Validation chromosome** | **r, source H1-hESC** | **r, source GM12878** | **r, source IMR-90** | **r, source K562** | **Selected source** |
| --- | --- | --- | --- | --- | --- | --- | --- |
| H1-hESC | 1 | 14 | — | 0.597 | 0.331 | 0.597 | GM12878 |
| H1-hESC | 2 | 15 | — | 0.580 | 0.359 | 0.590 | K562 |
| H1-hESC | 3 | 16 | — | 0.576 | 0.417 | 0.457 | GM12878 |
| H1-hESC | 4 | 13 | — | 0.657 | 0.322 | 0.597 | GM12878 |
| H1-hESC | 5 | 17 | — | 0.597 | 0.406 | 0.538 | GM12878 |
| GM12878 | 1 | 14 | 0.640 | — | 0.648 | 0.627 | IMR-90 |
| GM12878 | 2 | 15 | 0.601 | — | 0.587 | 0.627 | K562 |
| GM12878 | 3 | 16 | 0.690 | — | 0.632 | 0.687 | H1-hESC |
| GM12878 | 4 | 13 | 0.541 | — | 0.620 | 0.600 | IMR-90 |
| GM12878 | 5 | 17 | 0.651 | — | 0.633 | 0.703 | K562 |
| IMR-90 | 1 | 14 | 0.776 | 0.814 | — | 0.741 | GM12878 |
| IMR-90 | 2 | 15 | 0.735 | 0.798 | — | 0.748 | GM12878 |
| IMR-90 | 3 | 16 | 0.794 | 0.825 | — | 0.736 | GM12878 |
| IMR-90 | 4 | 13 | 0.804 | 0.786 | — | 0.792 | H1-hESC |
| IMR-90 | 5 | 17 | 0.780 | 0.793 | — | 0.725 | GM12878 |
| K562 | 1 | 14 | 0.180 | 0.632 | 0.501 | — | GM12878 |
| K562 | 2 | 15 | 0.505 | 0.654 | 0.324 | — | GM12878 |
| K562 | 3 | 16 | 0.620 | 0.689 | 0.107 | — | GM12878 |
| K562 | 4 | 13 | 0.251 | 0.210 | 0.119 | — | H1-hESC |
| K562 | 5 | 17 | 0.520 | 0.641 | 0.120 | — | GM12878 |

# Supplementary Table S9

Supplementary Table S9. Within-cell out-of-fold performance under chromosome-disjoint five-fold evaluation. All columns except the explicitly labelled prediction-averaged correlation are seedwise means. The calibration slope is the ordinary-least-squares slope of measured on predicted insulation; the reverse slope is that of predicted on measured. Depth recovery is the mean predicted value divided by the mean measured value over the 1% of loci with the lowest measured insulation.

| **Cell line** | **Bins scored** | **Pearson r (seedwise mean)** | **SD over seeds** | **r of the three-seed averaged prediction** | **Calibration slope (measured on predicted)** | **Reverse slope (predicted on measured)** | **R^2^ against the identity line** | **Depth recovery, lowest 1% of measured insulation** | **Loci in the lowest 1%** | **Per-chromosome range** | **Lowest chromosome** |
| --- | --- | --- | --- | --- | --- | --- | --- | --- | --- | --- | --- |
| H1-hESC | 241,078 | 0.791 | 0.0014 | 0.806 | 0.946 | 0.662 | 0.623 | 62.5% | 2,410 | 0.708–0.835 | chr19 |
| GM12878 | 244,234 | 0.780 | 0.0001 | 0.795 | 0.948 | 0.641 | 0.606 | 63.3% | 2,442 | 0.740–0.822 | chr4 |
| IMR-90 | 243,101 | 0.860 | 0.0030 | 0.869 | 0.914 | 0.809 | 0.730 | 71.0% | 2,431 | 0.821–0.906 | chr19 |
| K562 | 244,756 | 0.774 | 0.0041 | 0.788 | 0.899 | 0.667 | 0.591 | 52.7% | 2,447 | 0.661–0.864 | chr22 |

# Supplementary Table S10

Supplementary Table S10. Scale-resolved correlation on the fine-scale component. Seedwise-mean Pearson correlation between the measured and predicted fine-scale components at moving-average half-widths of 100, 250 and 500 kb (Materials and methods), the SD over seeds, and at 250 kb the difference from EpiInsula with its 95% union block-bootstrap interval and Benjamini–Hochberg-adjusted two-sided p. Differences are computed before rounding.

| **Cell line** | **Method** | **r, 100-kb half-width** | **SD** | **r, 250-kb half-width** | **SD** | **r, 500-kb half-width** | **SD** | **Δr versus EpiInsula at 250 kb** | **95% union block-bootstrap interval** | **BH-adjusted two-sided p** |
| --- | --- | --- | --- | --- | --- | --- | --- | --- | --- | --- |
| H1-hESC | EpiInsula | 0.804 | 0.0005 | 0.798 | 0.0009 | 0.797 | 0.0008 | — | — | — |
| H1-hESC | CT-TADB | 0.758 | 0.0038 | 0.745 | 0.0026 | 0.740 | 0.0031 | 0.053 | [0.048, 0.057] | <0.001 |
| H1-hESC | preciseTAD | 0.522 | 0.0008 | 0.517 | 0.0003 | 0.500 | 0.0007 | 0.281 | [0.274, 0.288] | <0.001 |
| H1-hESC | pTADS | 0.533 | 0.0002 | 0.523 | 0.0002 | 0.512 | 0.0002 | 0.275 | [0.268, 0.282] | <0.001 |
| H1-hESC | MLP | 0.604 | 0.0004 | 0.625 | 0.0008 | 0.627 | 0.0009 | 0.173 | [0.166, 0.180] | <0.001 |
| H1-hESC | SimpleCNN | 0.529 | 0.0009 | 0.541 | 0.0009 | 0.534 | 0.0006 | 0.257 | [0.253, 0.262] | <0.001 |
| H1-hESC | XGBoost | 0.536 | 0.0009 | 0.531 | 0.0004 | 0.518 | 0.0006 | 0.267 | [0.260, 0.274] | <0.001 |
| H1-hESC | Ridge | 0.496 | 0.0000 | 0.474 | 0.0000 | 0.473 | 0.0000 | 0.324 | [0.317, 0.332] | <0.001 |
| GM12878 | EpiInsula | 0.778 | 0.0018 | 0.774 | 0.0009 | 0.771 | 0.0008 | — | — | — |
| GM12878 | CT-TADB | 0.736 | 0.0232 | 0.732 | 0.0201 | 0.727 | 0.0202 | 0.042 | [0.038, 0.046] | <0.001 |
| GM12878 | preciseTAD | 0.446 | 0.0008 | 0.471 | 0.0004 | 0.461 | 0.0007 | 0.303 | [0.294, 0.312] | <0.001 |
| GM12878 | pTADS | 0.450 | 0.0014 | 0.471 | 0.0005 | 0.462 | 0.0002 | 0.303 | [0.294, 0.312] | <0.001 |
| GM12878 | MLP | 0.555 | 0.0008 | 0.572 | 0.0012 | 0.573 | 0.0020 | 0.202 | [0.193, 0.211] | <0.001 |
| GM12878 | SimpleCNN | 0.452 | 0.0013 | 0.476 | 0.0008 | 0.470 | 0.0007 | 0.298 | [0.292, 0.304] | <0.001 |
| GM12878 | XGBoost | 0.466 | 0.0019 | 0.488 | 0.0012 | 0.479 | 0.0004 | 0.286 | [0.277, 0.294] | <0.001 |
| GM12878 | Ridge | 0.365 | 0.0000 | 0.377 | 0.0000 | 0.373 | 0.0000 | 0.397 | [0.386, 0.407] | <0.001 |
| IMR-90 | EpiInsula | 0.855 | 0.0010 | 0.861 | 0.0008 | 0.860 | 0.0009 | — | — | — |
| IMR-90 | CT-TADB | 0.809 | 0.0064 | 0.811 | 0.0050 | 0.802 | 0.0052 | 0.050 | [0.047, 0.053] | <0.001 |
| IMR-90 | preciseTAD | 0.534 | 0.0003 | 0.574 | 0.0005 | 0.567 | 0.0003 | 0.287 | [0.280, 0.295] | <0.001 |
| IMR-90 | pTADS | 0.560 | 0.0002 | 0.591 | 0.0003 | 0.582 | 0.0002 | 0.270 | [0.263, 0.277] | <0.001 |
| IMR-90 | MLP | 0.674 | 0.0003 | 0.712 | 0.0007 | 0.717 | 0.0011 | 0.149 | [0.143, 0.155] | <0.001 |
| IMR-90 | SimpleCNN | 0.540 | 0.0014 | 0.580 | 0.0017 | 0.583 | 0.0012 | 0.281 | [0.275, 0.287] | <0.001 |
| IMR-90 | XGBoost | 0.574 | 0.0010 | 0.612 | 0.0007 | 0.605 | 0.0009 | 0.249 | [0.243, 0.255] | <0.001 |
| IMR-90 | Ridge | 0.484 | 0.0000 | 0.505 | 0.0000 | 0.503 | 0.0000 | 0.356 | [0.347, 0.365] | <0.001 |
| K562 | EpiInsula | 0.807 | 0.0021 | 0.801 | 0.0025 | 0.794 | 0.0024 | — | — | — |
| K562 | CT-TADB | 0.720 | 0.0078 | 0.707 | 0.0082 | 0.689 | 0.0084 | 0.094 | [0.087, 0.100] | <0.001 |
| K562 | preciseTAD | 0.514 | 0.0030 | 0.518 | 0.0017 | 0.492 | 0.0007 | 0.283 | [0.274, 0.291] | <0.001 |
| K562 | pTADS | 0.523 | 0.0009 | 0.514 | 0.0006 | 0.495 | 0.0004 | 0.287 | [0.279, 0.295] | <0.001 |
| K562 | MLP | 0.587 | 0.0030 | 0.601 | 0.0035 | 0.596 | 0.0027 | 0.200 | [0.192, 0.209] | <0.001 |
| K562 | SimpleCNN | 0.521 | 0.0038 | 0.532 | 0.0054 | 0.520 | 0.0056 | 0.269 | [0.264, 0.274] | <0.001 |
| K562 | XGBoost | 0.513 | 0.0010 | 0.511 | 0.0007 | 0.492 | 0.0007 | 0.290 | [0.280, 0.301] | <0.001 |
| K562 | Ridge | 0.469 | 0.0000 | 0.436 | 0.0000 | 0.424 | 0.0000 | 0.365 | [0.355, 0.374] | <0.001 |

# Supplementary Table S11

Supplementary Table S11. Regression against a classification twin. The classification twin is the unchanged network fitted on binarised boundary labels with a weighted binary cross-entropy loss. Boundary F1 uses validation-selected, test-frozen prominence; average precision ranks all boundary candidates by prominence. For the classification twin the correlation column is that of its logit track with the measured insulation score. Correlations of the regression model are seedwise means of the pooled decoded held-out series, as in Supplementary Table S9, with SD over three seeds. Refitting the regression model with the coefficient of the correlation term of the loss set to 0 (60 fits) changes no entry of the regression rows by more than 0.004 (correlation by at most 0.003, boundary F1 by at most 0.004 within ±10 kb and 0.002 within ±20 kb, average precision by at most 0.004); the per-seed ranges of the two fits overlap for every cell line and endpoint.

| **Cell line** | **Model** | **Pearson r with measured insulation (SD)** | **Boundary F1 ±10 kb (SD)** | **Boundary F1 ±20 kb (SD)** | **Average precision over boundary candidates (SD)** |
| --- | --- | --- | --- | --- | --- |
| H1-hESC | Regression (reported model) | 0.791 (0.0014) | 0.594 (0.0026) | 0.692 (0.0009) | 0.596 (0.0067) |
| H1-hESC | Classification twin | −0.357 (0.0025) [logit track] | 0.506 (0.0066) | 0.564 (0.0065) | 0.492 (0.0058) |
| GM12878 | Regression (reported model) | 0.780 (0.0001) | 0.500 (0.0045) | 0.612 (0.0027) | 0.504 (0.0049) |
| GM12878 | Classification twin | −0.397 (0.0075) [logit track] | 0.404 (0.0020) | 0.464 (0.0036) | 0.371 (0.0048) |
| IMR-90 | Regression (reported model) | 0.860 (0.0030) | 0.597 (0.0055) | 0.699 (0.0036) | 0.614 (0.0065) |
| IMR-90 | Classification twin | −0.469 (0.0047) [logit track] | 0.508 (0.0101) | 0.560 (0.0101) | 0.496 (0.0142) |
| K562 | Regression (reported model) | 0.774 (0.0041) | 0.540 (0.0038) | 0.648 (0.0024) | 0.537 (0.0043) |
| K562 | Classification twin | −0.416 (0.0044) [logit track] | 0.471 (0.0021) | 0.536 (0.0050) | 0.463 (0.0132) |

# Supplementary Table S12

Supplementary Table S12. Separation of Strong from Weak reference boundaries by insulation depth. Area under the receiver operating characteristic curve for classifying the 4D Nucleome Strong and Weak calls that fall on evaluated 10-kb bins by the predicted (seedwise mean, SD over seeds) and by the measured insulation depth at the boundary bin.

| **Cell line** | **Strong reference boundaries** | **Weak reference boundaries** | **AUC, predicted depth (SD over seeds)** | **AUC, measured depth** |
| --- | --- | --- | --- | --- |
| H1-hESC | 6,235 | 3,550 | 0.750 (0.0042) | 0.897 |
| GM12878 | 4,486 | 3,873 | 0.743 (0.0043) | 0.859 |
| IMR-90 | 5,143 | 2,787 | 0.699 (0.0069) | 0.793 |
| K562 | 4,109 | 3,442 | 0.690 (0.0093) | 0.794 |

# Supplementary Table S13

Supplementary Table S13. Leave-one-track-out effects. Changes in held-out Pearson correlation are seedwise means with unadjusted 95% gap-aware 200-bin block-bootstrap intervals and fold sign counts.

| **Cell line** | **Full six-track held-out r** | **Δr on removing CTCF** | **Δr on removing RAD21** | **Δr on removing ATAC-seq** | **Δr on removing histones** |
| --- | --- | --- | --- | --- | --- |
| H1-hESC | 0.791 | −0.015 [−0.017, −0.012] (5/5) | −0.068 [−0.072, −0.065] (5/5) | −0.003 [−0.005, −0.001] (4/5) | −0.031 [−0.034, −0.027] (5/5) |
| GM12878 | 0.780 | −0.021 [−0.024, −0.018] (5/5) | −0.019 [−0.022, −0.017] (5/5) | −0.013 [−0.015, −0.011] (5/5) | −0.024 [−0.028, −0.021] (5/5) |
| IMR-90 | 0.860 | 0.000 [−0.001, +0.001] (4/5) | −0.008 [−0.010, −0.006] (5/5) | −0.012 [−0.014, −0.010] (5/5) | −0.032 [−0.035, −0.029] (5/5) |
| K562 | 0.774 | −0.012 [−0.016, −0.009] (5/5) | −0.034 [−0.037, −0.030] (5/5) | −0.008 [−0.011, −0.004] (4/5) | −0.040 [−0.045, −0.035] (5/5) |
| Mean of the four cell lines | 0.801 | −0.012 [−0.013, −0.011] | −0.032 [−0.034, −0.031] | −0.009 [−0.010, −0.008] | −0.032 [−0.034, −0.030] |

# Supplementary Table S14

Supplementary Table S14. Threshold-grid robustness of the IMR-90/K562 cell-type-specific recovery endpoint. The two measured thresholds (−1.5 and −0.3) were scaled jointly, and the two predicted thresholds (−1.0 and −0.5) jointly, each by 0.8, 0.9, 1.0, 1.1 or 1.2; the unscaled point reproduces Figure 7E. Columns give the size of the contrasting set, the recovered fraction for each method (seedwise mean), the two highest-ranked methods and their margin, computed before rounding.

| **Measured-threshold scale** | **Predicted-threshold scale** | **Contrasting loci** | **EpiInsula** | **CT-TADB** | **preciseTAD** | **pTADS** | **MLP** | **SimpleCNN** | **XGBoost** | **Ridge** | **Highest** | **Second** | **Margin** |
| --- | --- | --- | --- | --- | --- | --- | --- | --- | --- | --- | --- | --- | --- |
| 0.8 | 0.8 | 2,961 | 0.419 | 0.135 | 0.081 | 0.074 | 0.297 | 0.110 | 0.129 | 0.089 | EpiInsula | MLP | 0.122 |
| 0.8 | 0.9 | 2,961 | 0.387 | 0.091 | 0.049 | 0.036 | 0.248 | 0.078 | 0.091 | 0.059 | EpiInsula | MLP | 0.138 |
| 0.8 | 1.0 | 2,961 | 0.354 | 0.062 | 0.028 | 0.015 | 0.211 | 0.062 | 0.061 | 0.036 | EpiInsula | MLP | 0.144 |
| 0.8 | 1.1 | 2,961 | 0.320 | 0.037 | 0.015 | 0.010 | 0.182 | 0.050 | 0.040 | 0.019 | EpiInsula | MLP | 0.138 |
| 0.8 | 1.2 | 2,961 | 0.281 | 0.022 | 0.008 | 0.005 | 0.152 | 0.043 | 0.029 | 0.011 | EpiInsula | MLP | 0.129 |
| 0.9 | 0.8 | 2,069 | 0.425 | 0.143 | 0.086 | 0.075 | 0.311 | 0.110 | 0.127 | 0.088 | EpiInsula | MLP | 0.114 |
| 0.9 | 0.9 | 2,069 | 0.404 | 0.102 | 0.056 | 0.041 | 0.269 | 0.081 | 0.096 | 0.059 | EpiInsula | MLP | 0.135 |
| 0.9 | 1.0 | 2,069 | 0.384 | 0.071 | 0.035 | 0.019 | 0.239 | 0.067 | 0.071 | 0.038 | EpiInsula | MLP | 0.146 |
| 0.9 | 1.1 | 2,069 | 0.357 | 0.046 | 0.020 | 0.012 | 0.211 | 0.057 | 0.047 | 0.022 | EpiInsula | MLP | 0.147 |
| 0.9 | 1.2 | 2,069 | 0.323 | 0.029 | 0.011 | 0.007 | 0.182 | 0.050 | 0.034 | 0.015 | EpiInsula | MLP | 0.141 |
| 1.0 | 0.8 | 1,510 | 0.409 | 0.148 | 0.088 | 0.074 | 0.328 | 0.108 | 0.126 | 0.089 | EpiInsula | MLP | 0.081 |
| 1.0 | 0.9 | 1,510 | 0.396 | 0.108 | 0.059 | 0.043 | 0.290 | 0.079 | 0.097 | 0.060 | EpiInsula | MLP | 0.106 |
| 1.0 | 1.0 | 1,510 | 0.383 | 0.081 | 0.036 | 0.024 | 0.264 | 0.069 | 0.073 | 0.042 | EpiInsula | MLP | 0.119 |
| 1.0 | 1.1 | 1,510 | 0.374 | 0.052 | 0.021 | 0.014 | 0.234 | 0.060 | 0.050 | 0.026 | EpiInsula | MLP | 0.141 |
| 1.0 | 1.2 | 1,510 | 0.346 | 0.035 | 0.010 | 0.008 | 0.206 | 0.056 | 0.036 | 0.017 | EpiInsula | MLP | 0.140 |
| 1.1 | 0.8 | 1,149 | 0.386 | 0.156 | 0.077 | 0.067 | 0.312 | 0.105 | 0.117 | 0.086 | EpiInsula | MLP | 0.075 |
| 1.1 | 0.9 | 1,149 | 0.382 | 0.121 | 0.062 | 0.038 | 0.288 | 0.082 | 0.095 | 0.058 | EpiInsula | MLP | 0.094 |
| 1.1 | 1.0 | 1,149 | 0.377 | 0.094 | 0.038 | 0.026 | 0.264 | 0.075 | 0.075 | 0.044 | EpiInsula | MLP | 0.113 |
| 1.1 | 1.1 | 1,149 | 0.375 | 0.063 | 0.024 | 0.017 | 0.239 | 0.067 | 0.055 | 0.033 | EpiInsula | MLP | 0.136 |
| 1.1 | 1.2 | 1,149 | 0.360 | 0.044 | 0.012 | 0.010 | 0.214 | 0.065 | 0.042 | 0.023 | EpiInsula | MLP | 0.146 |
| 1.2 | 0.8 | 890 | 0.349 | 0.158 | 0.070 | 0.060 | 0.286 | 0.097 | 0.110 | 0.071 | EpiInsula | MLP | 0.063 |
| 1.2 | 0.9 | 890 | 0.357 | 0.124 | 0.059 | 0.032 | 0.272 | 0.080 | 0.089 | 0.047 | EpiInsula | MLP | 0.085 |
| 1.2 | 1.0 | 890 | 0.356 | 0.104 | 0.037 | 0.021 | 0.259 | 0.078 | 0.072 | 0.039 | EpiInsula | MLP | 0.097 |
| 1.2 | 1.1 | 890 | 0.363 | 0.072 | 0.024 | 0.015 | 0.240 | 0.075 | 0.055 | 0.033 | EpiInsula | MLP | 0.123 |
| 1.2 | 1.2 | 890 | 0.359 | 0.053 | 0.012 | 0.010 | 0.220 | 0.078 | 0.043 | 0.024 | EpiInsula | MLP | 0.139 |

# Supplementary Table S15

Supplementary Table S15. Cell-type-specific recovery endpoint for all six cell-line pairs. For each pair, with the contrasting set pooled over both directions, the table gives the size of the contrasting set and the recovered fraction of every method at the unscaled thresholds (seedwise means), and over the 25-point threshold grid the range of contrasting-set sizes, the range of EpiInsula’s recovered fraction, the number of grid points at which EpiInsula ranks first, the second-ranked method with its number of points, and the range of the margin over the second-ranked method, computed before rounding.

| **Cell-line pair** | **Contrasting loci (unscaled thresholds)** | **EpiInsula recovered fraction** | **CT-TADB recovered fraction** | **preciseTAD recovered fraction** | **pTADS recovered fraction** | **MLP recovered fraction** | **SimpleCNN recovered fraction** | **XGBoost recovered fraction** | **Ridge recovered fraction** | **Contrasting loci over the grid** | **EpiInsula range over the grid** | **Points at which EpiInsula ranks first** | **Second-ranked method (points)** | **Margin range** |
| --- | --- | --- | --- | --- | --- | --- | --- | --- | --- | --- | --- | --- | --- | --- |
| H1-hESC / GM12878 | 1,482 | 0.378 | 0.144 | 0.074 | 0.065 | 0.244 | 0.132 | 0.116 | 0.059 | 703–3,159 | 0.240–0.422 | 25/25 | MLP (25) | 0.036–0.157 |
| H1-hESC / IMR-90 | 1,514 | 0.435 | 0.128 | 0.035 | 0.023 | 0.257 | 0.071 | 0.075 | 0.038 | 616–3,836 | 0.252–0.508 | 25/25 | MLP (25) | 0.139–0.224 |
| H1-hESC / K562 | 2,017 | 0.356 | 0.102 | 0.063 | 0.069 | 0.193 | 0.161 | 0.097 | 0.103 | 1,117–4,156 | 0.230–0.397 | 25/25 | MLP (20), SimpleCNN (5) | 0.105–0.194 |
| GM12878 / IMR-90 | 1,145 | 0.430 | 0.152 | 0.025 | 0.013 | 0.283 | 0.046 | 0.056 | 0.026 | 493–3,215 | 0.217–0.510 | 25/25 | MLP (25) | 0.079–0.168 |
| GM12878 / K562 | 1,955 | 0.343 | 0.117 | 0.045 | 0.043 | 0.207 | 0.114 | 0.054 | 0.073 | 1,148–4,036 | 0.201–0.416 | 25/25 | MLP (25) | 0.081–0.178 |
| IMR-90 / K562 | 1,510 | 0.383 | 0.081 | 0.036 | 0.024 | 0.264 | 0.069 | 0.073 | 0.042 | 890–2,961 | 0.281–0.425 | 25/25 | MLP (25) | 0.063–0.147 |

# Supplementary Figure S1

Supplementary Figure S1. Boundary F1 at three matching tolerances. For each cell line, points give the seedwise-mean F1 of EpiInsula and the seven comparison methods against the 4D Nucleome reference calls (Materials and methods) when a predicted boundary must fall on the exact reference bin, within ±10 kb or within ±20 kb. The prominence is that of Supplementary Table S7; the tolerance changes only the matching. The printed value is EpiInsula’s margin over the second-ranked method. Error bars on EpiInsula are ± SD over three seeds; comparison methods are plotted as seedwise means.

*Alt text:* Three dot panels give boundary F1 for EpiInsula and seven comparison methods in four cell lines at exact-bin, ±10-kb and ±20-kb matching, with EpiInsula highest in every panel.

# Supplementary Figure S2

Supplementary Figure S2. Contact-map prediction methods retrained on the same data, one window per cell line. (A–D) For H1-hESC, GM12878, IMR-90 and K562, a 2.1-Mb window on a fold-1 test chromosome centred on the deepest measured boundary of that chromosome, chosen from the measured insulation score alone and at least 2 Mb from the chromosome ends. Rows: the measured Hi-C matrix and the maps predicted by Epiphany, ChromNet and C.Origami after retraining (each map scaled to its own 98th percentile; Epiphany predicts only the ±1-Mb diagonal band); the measured insulation score with the EpiInsula prediction; and the insulation scores derived from the three predicted maps. Correlations in the legends are pooled over the five fold-1 test chromosomes at seed 42.

*Alt text:* Four genome-browser blocks, one per cell line, each stacking a measured Hi-C matrix, three predicted matrices and insulation-score tracks from EpiInsula and from the three contact-map predictors.

# Supplementary Figure S3

Supplementary Figure S3. Scale-resolved correlation. (A) Seedwise-mean Pearson correlation between the fine-scale components of measured and predicted insulation at moving-average half-widths of 100, 250 and 500 kb, for EpiInsula and seven comparison methods in four cell lines; error bars are ± SD over three seeds. (B) Difference in fine-scale correlation between EpiInsula and each comparison method at the 250-kb half-width with 95% union block-bootstrap intervals over 200-, 500- and 1,000-bin blocks; all 28 intervals exclude zero.

*Alt text:* Grouped dot chart of fine-scale correlation at three half-widths for eight methods in four cell lines, and a forest plot of the 28 differences from EpiInsula, all above zero.

# Supplementary Figure S4

Supplementary Figure S4. Leave-one-track-out effects per fold. Grey points are fold-specific changes; navy points and intervals are pooled estimates with unadjusted 95% gap-aware 200-bin block-bootstrap intervals. CTCF in IMR-90 is shown in grey because its interval includes zero.

*Alt text:* Dot-and-interval chart of the change in held-out correlation after removing each input group, one panel per cell line and one row per input group, with grey per-fold points and navy pooled estimates.

# Supplementary Figure S5

Supplementary Figure S5. Input removal and dose response. The two descriptive summaries compare mean unsigned leave-one-track-out change with the fraction of cell lines showing increasing response magnitude on both scaling branches. These summaries are reported without inferential intervals or hypothesis tests.

*Alt text:* Two-point scatter relating the mean input-removal effect of CTCF and RAD21 to the fraction of cell lines with a graded dose response.

# Supplementary Figure S6

Supplementary Figure S6. Threshold-grid robustness of the IMR-90/K562 recovery endpoint. (A) Fraction of contrasting loci recovered by EpiInsula when the two measured thresholds and, independently, the two predicted thresholds are scaled jointly by 0.8 to 1.2; the outlined cell is the unscaled point reported in Figure 7E. (B) Margin between EpiInsula and the second-ranked method at the same points; the second-ranked method is the multilayer perceptron throughout.

*Alt text:* Two five-by-five heat maps over scaled contrast and recovery thresholds showing the recovered fraction for EpiInsula and its margin over the second-ranked method, with the unscaled cell outlined.
